# Comparing Classical and Machine Learning Force Fields for Modeling Deformation of Metal–Organic Frameworks Relevant for Direct Air Capture

**DOI:** 10.1021/acs.jpcc.5c04020

**Published:** 2025-09-08

**Authors:** Logan M. Brabson, Andrew J. Medford, David S. Sholl

**Affiliations:** † School of Chemical and Biomolecular Engineering, 332139 Georgia Institute of Technology, Atlanta, Georgia 30332, United States; ‡ 6146 Oak Ridge National Laboratory, Oak Ridge, Tennessee 37830, United States

## Abstract

Deformation of metal–organic frameworks (MOFs) induced by adsorbate molecules can affect adsorption properties such as capacity and selectivity, but most computational studies of MOFs assume framework rigidity to simplify calculations. Although flexible force fields (FFs) for MOFs have been parametrized for specific materials, the generality of FFs for reliably modeling adsorbate-induced deformation to accuracy nearing that of density functional theory (DFT) has not been established. This work confirms using DFT calculations that adsorbate-induced deformation can affect CO_2_ and H_2_O adsorption energies in a considerable fraction of MOFs promising for direct air capture (DAC). We then benchmark the efficacy of several general-purpose FFs in describing adsorbate-induced deformation for DAC against DFT. Our results show that current classical FFs are insufficient for describing MOF deformation, especially in cases of interest for DAC where strong interactions exist between adsorbed molecules and MOF frameworks. Some emerging machine learning force fields (MLFFs) we tested, particularly CHGNet, MACE-MP-0, and Equiformer V2, appear to be more promising than the classical FF for emulating the deformation behavior described by DFT. The best performing FF (CHGNet), however, fails to achieve the accuracy required for practical predictions with a mean absolute adsorption energy error of 0.124 eV.

## Graphical abstract

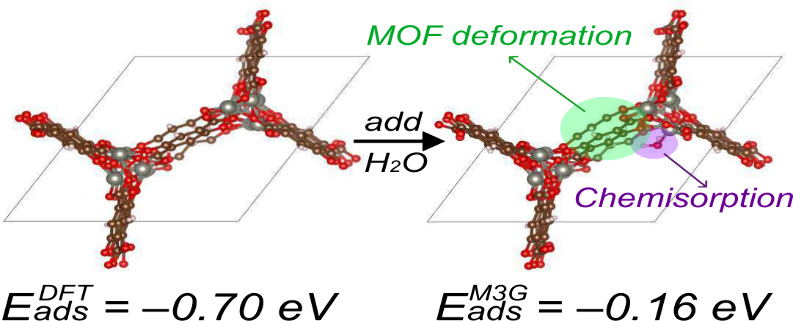

## Introduction

Direct air capture (DAC) seeks to capture large quantities of carbon dioxide directly from atmospheric air but remains a challenging scientific problem due to both the low partial pressure of CO_2_ in the air and the need to identify adsorbents suitable for repeated use in cost-effective processes.^1–4^ Metal–organic frameworks (MOFs) are a promising class of solid sorbents for DAC at low temperatures given their high porosities, modularity, and tunability.^5–7^ In contrast with more traditional liquid DAC sorbents such as alkali hydroxides, MOFs can bind CO_2_ via physisorption in addition to chemisorption and typically require less energy for regeneration.^8,9^

One important consideration when using MOFs for gas separations is their flexibility.^10–12^ Framework flexibility can be either intrinsic or induced by guest molecules. Intrinsic flexibility exists in all MOFs as a result of thermal vibrations, whereas adsorbate-induced deformation only occurs in some MOFs.^13^ Many MOFs exhibit dynamic porosity,^14^ such as breathing, swelling,^15,16^ or gating,^17^ in response to external stimuli. Adsorbate-induced deformation can also manifest as more subtle changes such as linker rotation.^18^ Local deformations induced by adsorbates are often ignored in modeling. Accounting for flexibility of any nature makes computational models more physically accurate, and doing so indeed has a non-negligible influence on properties of interest.^19^

Despite flexibility playing a role in accurate modeling of adsorption, almost all large-scale computational studies of MOFs for gas separations assume MOF rigidity and only consider nonbonded interactions during molecular dynamics or Monte Carlo simulations.^20–23^ Yu et al. showed that molecular simulations performed with this rigidity assumption can cause significant underestimations of gas uptake for a variety of physisorbed molecules in MOFs at dilute conditions and proposed several possible strategies for approximating framework flexibility in molecular simulations.^24^ These and related strategies, however, suffer from either insufficient accuracy or prohibitively high computational cost for high-throughput studies.^25^ Unfortunately, the ability of common models such as classical force fields to adequately describe MOFs with intrinsic flexibility^11,13,26^ or MOFs that undergo structural deformation in the presence of guest molecules (adsorbate-induced deformation)^18,27^ is not well established.

We limit our focus to adsorbate-induced MOF deformations in this work because such deformations are common in modeling many MOFs, including those not known to experience other modes of flexibility. An example of a MOF that undergoes adsorbate-induced deformation from the large collection of DFT results from the recent Open DAC 2023 work^28^ can be seen in Figure 1. In this MOF, the introduction of a H_2_O guest molecule, shaded in purple, yields a relaxed structure with the linker, shaded in green, expanded outward relative to its position in the empty MOF. Without the H_2_O molecule included, the MOF structure on the right is more than 0.04 eV/linker (0.3 eV/unit cell) less favorable. Across all seven linker molecules in the unit cell, this 0.3 eV deformation energy is significant relative to adsorption energies that typically range from 0 to 1 eV. Although the visual changes in geometry are minor, these slight changes in atomic positions can add up over the 100+ atoms in a typical MOF unit cell.

**Figure 1:**
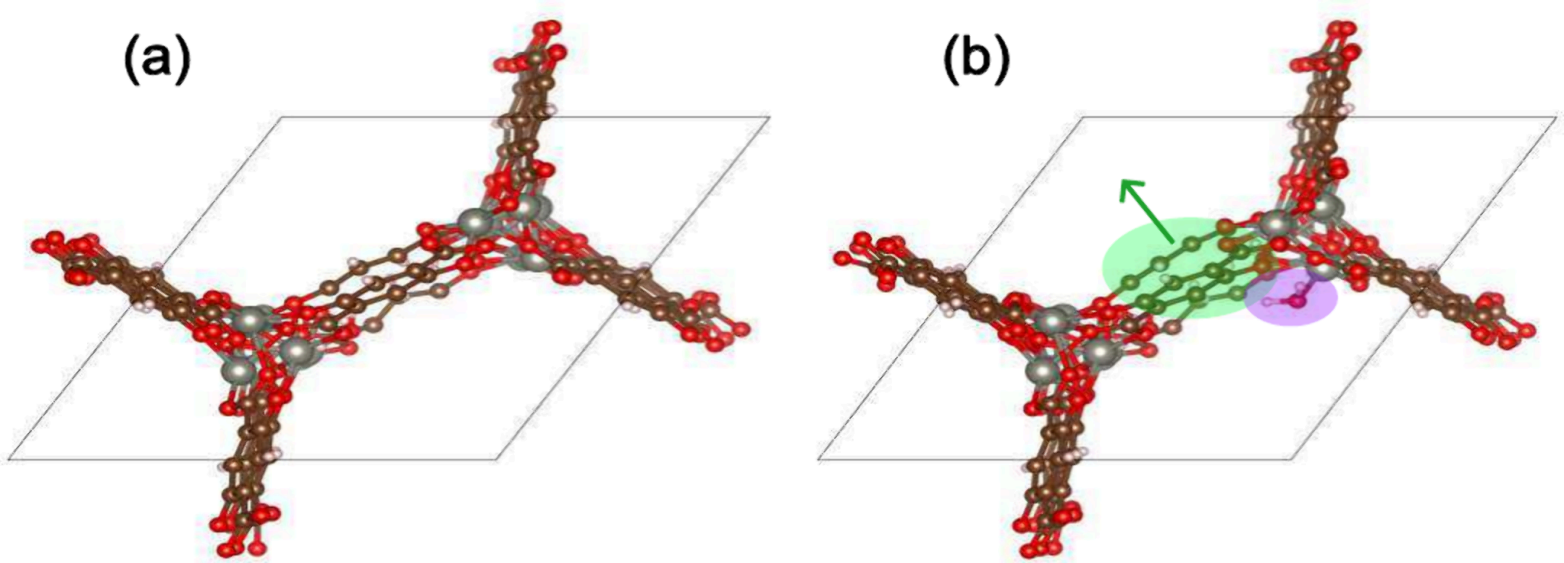
DFT-relaxed structures for the defective MOF with CSD code WOBHEB (system ID 0_217) (a) before and (b) after the introduction of one H_2_O adsorbate molecule, shaded in purple. The green arrow indicates the expansion of the linker upon adsorption. Zn, C, O, and H atoms are shown as silver, brown, red, and white, respectively.

The vast number of possible MOFs implies that computation can play an important role in screening materials, provided the underlying models have sufficient accuracy.^29^ The high computational cost of quantum mechanical (QM) calculations such as density functional theory (DFT) necessitates the use of force fields (FFs) to improve efficiency of high-throughput materials screening (HTS) studies.^30–35^ FFs have been developed to account for MOF flexibility, including adsorbate-induced deformation, but they are typically system-specific and exhibit poor transferability.^36,37^ Machine learning force fields (MLFFs) trained on QM data can bridge the gap between classical FFs and ab initio methods, but they must be applicable beyond their underlying training data to be used widely.^38,39^ A wide variety of ML approaches have been applied to help identify ideal adsorbents for CO_2_ capture.^40^ Some emerging “foundational” MLFFs based on deep graph neural networks seek to universally describe the entire periodic table, such as the Materials Graph Network with three-body interactions (M3GNet),^41,42^ Crystal Hamiltonian Graph Neural Network (CHGNet),^43^ and the Multi-Atomic Cluster Expansion (MACE)^44,45^ architecture.

Batatia et al. released several pretrained MACE models called MACE-MP-0, and they showed that the (medium-sized) MACE-MP-0 MLFF was widely applicable to atomic structures for catalysis and to aqueous systems.^46^ MACE-MP-0 performs well at predicting MOF energies for ≥20,000 DFT-relaxed MOFs from the Quantum MOF database^47^ and at describing CO_2_ adsorption in Mg-MOF-74. M3GNet, CHGNet, and MACE-MP-0 were trained on the Materials Project (MP) database of inorganic crystals.^48^ The CHGNet and MACE-MP-0 training sets are an order of magnitude larger than that of M3GNet because CHGNet and MACE-MP-0 were trained on the MPtrj subset of the MP database, which includes structural relaxation trajectories for the MP materials.^43^ While the MP database contains a large number of solids, it is biased heavily toward oxides and contains relatively few MOFs. Batatia et al. released an updated MACE model called MACE-MPA-0 trained on 3.5 million structures from the MPtrj and sAlex databases. The sAlex data set was developed as part of the Open Materials 2024 project.^49,50^ MACE-MPA-0 outperforms MACE-MP-0 on Matbench benchmarks,^51^ though the authors caution that improved accuracy is not guaranteed in all cases. Most recently, Fu et al. trained their equivariant Smooth Energy Network (eSEN) on the combined OMat, MPtrj, and sAlex data sets with an emphasis on conserving energy during molecular dynamics simulations. eSEN achieved state-of-the-art results on physical property predictions tasks including materials stability and thermal conductivity predictions.^52^ Table 1 shows the size of the training data set and the number of model parameters for the MLFFs considered in this study.

**Table 1:** Training Dataset Size, Model Size, and Force Consistency for the Six MLFFs Used in This Study^a^

model	# of training structures	# of parameters	force consistency
M3GNet^42^	188k	228k	Yes
CHGNet^43^	1.58M	413k	Yes
MACE-MP-0 (medium)^46^	1.58M	4.69M	Yes
MACE-MPA-0 (medium)^46^	11.98M	9.06M	Yes
eSEN-30M-OAM^52^	113M	30.2M	Yes
ODAC Equiformer V2 (large)^28^	38M	153M	No

a Force consistent models calculate forces as the gradient of the energy surface.

Recent work has made progress in assessing the accuracy of universal MLFFs for various chemical applications. Focassio et al. showed that three common MLFFs – MACE-MP-0, CHGNet,^43^ and M3GNet – did not adequately describe a set of 1,497 solid surfaces including 73 unique elements but that these models could be useful for fine-tuning more specialized models.^53^ For MOFs, Zheng et al. showed that molecular dynamics using the Deep Potential-Smooth Edition (DeepPot-SE)^54^ MLFF is more capable than UFF in describing Mg-MOF-74 flexibility, which enhances CO_2_ diffusivity in the pores compared to rigid models.^55^ Riebesell et al. published a leaderboard for ranking MLFFs by their ability to predict MOF thermodynamic stability, but no comparison to a classical FF was provided.^51^ They found that MLFFs trained on forces and stresses from DFT relaxation trajectories, including M3GNet and MACE, outperform MLFFs that do not. Despite these advances, no systematic comparison of classical and universal MLFFs has been performed for MOFs in the context of an application such as DAC.

This work builds upon the recent Open DAC 2023 (ODAC23) data set of more than 38 million DFT calculations for CO_2_ and H_2_O adsorption in MOFs.^28^ That work sought to raise the baseline level of theory for computational DAC studies in MOFs from classical FFs (e.g., UFF) to DFT. As might be expected, the accuracy of UFF compared to DFT results differs significantly between physisorbed and chemisorbed molecules. The ODAC23 project also trained the Equiformer architecture,^56^ previously used for the Open Catalyst project,^57,58^ to create a customized ML model capable of directly predicting the adsorption energy of CO_2_ or H_2_O in MOFs. Although this model outperformed UFF, a more thorough comparison of Equiformer’s performance relative to other MLFFs is needed, particularly in cases where deformation is significant.

In this paper, we selected a diverse set of 60 MOF+adsorbate systems from the ODAC Equiformer test set while prioritizing MOFs that are potentially favorable for DAC given their selectivity toward adsorption of CO_2_ over H_2_O according to ODAC23 DFT calculations.^59^ We begin by showing that adsorption energy calculations in rigid and deformable MOFs frequently differ according to DFT calculations, particularly when adsorbate molecules chemisorb. Taking DFT adsorption energies as the ground truth, we then compare a widely used classical FF (UFF4MOF, referred to as UFF in this work for simplicity)^60,61^ to M3GNet, CHGNet, eSEN, and the pretrained MACE-MP-0 and MACE-MPA-0 models. We also include the tailored Equiformer V2 (Large) model from ODAC23 (EqV2-ODAC) in our comparison.^28^ Three of the MLFFs, namely, CHGNet, MACE-MP-0, and EqV2-ODAC, outperform the classical approach in predicting adsorption energies in MOFs that undergo adsorbate-induced deformation. The relative success of CHGNet and MACE-MP-0 given their training data is surprising and deserves further study, but analysis of MLFF structural and chemical features leading to this result are beyond the scope of this work. Unfortunately, we do not identify a model that predicts a mean absolute adsorption energy error relative to DFT of less than 0.1 eV, a reasonable upper bound on errors for practical calculations. The findings suggest that accounting for adsorbate-induced MOF deformation is critical to achieve accurate chemisorption predictions but that additional force field development is needed to achieve this across all MOFs.

## Methods

### Energy Definitions

A key quantity in any description of molecular adsorption in MOFs is the adsorption energy for a single molecule, *E*_*ads*_. The adsorption energy is defined by

$$E_{ads}=E_{sys}\left(r_{sys}\right)-E_{MOF}\left(r_{MOF}\right)-E_{adsorbate}\left(r_{adsorbate}\right)$$ 1

where in *E*_*x*_(*r*_*y*_), *x* refers to the set of atoms considered in the energy calculation, with *y* defining the atoms included in the geometry used for relaxation. The subscript *sys* refers to a combined MOF+adsorbate geometry and the subscript *adsorbate* refers to the isolated gas-phase adsorbate. All energy terms are at relaxed geometries. In Figure 1, *r*_*MOF*_ and *r*_*sys*_ refer to the geometries shown in (a) and (b), respectively. *E*_*ads*_ is the primary metric of interest in this work, and a good FF for practical simulations should agree with DFT to within ± 0.1 eV. To highlight the influence of deformation of the MOF framework, it is useful to decompose *E*_*ads*_ into energies associated with MOF-molecule interactions (*E*_*int*_) and MOF deformation (*E*_*MOF*,*deform*_). As we will show, this decomposition is critical to deconvoluting the influence of MOF deformation, but we emphasize that the physically significant quantity is *E*_*ads*_.

We define the MOF-molecule interaction energy as

$$E_{int}=E_{sys}\left(r_{sys}\right)-E_{MOF}\left(r_{sys}\right)-E_{adsorbate}\left(r_{sys}\right)$$ 2

Unlike the adsorption energy, the geometry of the MOF used in every term in Eq. 2 is the same so that the interaction energy captures only the electronic contributions. Notably, *E*_*adsorbate*_(*r*_*sys*_) implies that periodic boundary conditions are used, which will remove any self-interactions between the adsorbates from *E*_*int*_. In practice, it is often more convenient to use nonperiodic vacuum conditions for the adsorbate, which introduces an adsorbate self-interaction energy:

$$E_{adsorbate,self}=E_{adsorbate}\left(r_{sys}\right)-E_{adsorbate}\left(r_{sys,vacuum}\right)$$ 3

*E*_*adsorbate*,*self*_ quantifies the energy that arises due to placing adsorbates in a periodic system and should be negligible in low loadings or for MOFs with large unit cells.

In a typical high-throughput molecular simulation assuming MOF rigidity, the position of framework atoms in *r*_*sys*_ are identical to the crystal structure of the empty MOF, so *r*_*sys*_ = *r*_*MOF*_ and *E*_*int*_ becomes equivalent to *E*_*ads*_ when *E*_*adsorbate*,*self*_ can be neglected. A more physically complete description of adsorption recognizes that the MOF geometry after adsorption is different than in the empty MOF structure, so *E*_*ads*_ is not equal to *E*_*int*_.

To quantify the contributions to adsorption due to MOF and adsorbate deformation, it is useful to define

$$E_{MOF,deform}=E_{MOF}\left(r_{sys}\right)-E_{MOF}\left(r_{MOF}\right)$$ 4

$$E_{adsorbate,deform}=E_{adsorbate}\left(r_{sys,vacuum}\right)-E_{adsorbate}\left(r_{adsorbate}\right)$$ 5

The energies on the right-hand sides of eqs 4 and 5 are for the same sets of atoms at different geometries. These energies describe the effects of atomic movements during adsorption for the MOF and adsorbate, respectively.

Equations 1–5 can be combined to show that the difference between the adsorption and interaction energies is equivalent to the sum of the MOF deformation, the adsorbate deformation, and the adsorbate self-interaction energy:

$$E_{ads}-E_{int}=E_{MOF,deform}+E_{adsorbate,deform}+E_{adsorbate,self}$$ 6

Most molecular simulations of small molecules treat the molecule as rigid such that the *E*_*adsorbate*,*deform*_ term in eq 6 is exactly zero. In DFT calculations, the molecule can deform, so this term cannot necessarily be omitted.

It is also useful to define an energy term representative of the binding energies used in molecular simulations where the MOF is held rigid. For example, consider a grand canonical Monte Carlo (GCMC) simulation to compute the uptake of a guest molecule in a MOF. The key quantity used to accept or reject trial moves in such simulations is the binding energy of the guest molecule to the framework, and almost all published GCMC simulations assume MOF rigidity. Thus, neither *E*_*ads*_ nor *E*_*int*_ are directly applicable to this situation because, in this work, we have relaxed the rigid MOF assumption and both *E*_*ads*_ and *E*_*int*_ explicitly allow for the movement of framework atoms upon adsorption. To disambiguate, we define the static adsorption energy as

$$E_{static}=E_{sys}\left(r_{MOF},r_{adsorbate}\right)-E_{MOF}\left(r_{MOF}\right)-E_{adsorbate}\left(r_{adsorbate}\right)$$ 7

*E*_*static*_ is analogous to *E*_*ads*_ with the MOF being held rigid during combined MOF+adsorbate relaxation. That is, *r*_*MOF*_ in the *E*_*sys*_ term is the geometry determined from empty MOF relaxation in the *E*_*MOF*_ term of eq 7, and only the adsorbate positions were allowed to change during system relaxation.

*E*_*static*_ can be considered a type of interaction energy since the MOF is held rigid during relaxation but differs from the definition of *E*_*int*_ in eq 2 because the MOF geometry for *E*_*static*_ is determined from relaxation of the empty MOF without a guest molecule. That is, *E*_*static*_ is equivalent to the adsorption (*E*_*ads*_) and interaction (*E*_*int*_) energies when the MOF is treated as rigid in MOF+adsorbate relaxations. We define *E*_*int*_ separately from *E*_*static*_ because *E*_*int*_ is used to conveniently decompose *E*_*ads*_ into MOF-adsorbate interactions and MOF deformation contributions, but *E*_*static*_ is the quantity used as the adsorption energy in the majority of the current literature on gas separations in MOFs.

### Energy Calculations

All adsorption energy calculations were performed in a similar manner as in ODAC23. Empty MOFs were first relaxed with the unit cell parameters included as degrees of freedom. Adsorbate molecules were then placed according to the ODAC23 placements, and the combined geometry was relaxed while holding cell parameters fixed to give *E*_*sys*_(*r*_*sys*_). The adsorbed-state MOF energy, *E*_*MOF*_(*r*_*sys*_), was then determined by removing the adsorbate from the combined system and computing the single point energy of the MOF. In a departure from the method used on ODAC23, we then relaxed the empty MOF starting from its adsorbed-state geometry using fixed unit cell dimensions. The resulting energy was compared to that from the original empty MOF relaxation, and the lower (more favorable) energy structure was taken to be the ground state reference MOF with energy *E*_*MOF*_(*r*_*MOF*_). Adsorbates were placed in the ground state MOF and relaxed while holding the MOF atom positions fixed for *E*_*static*_ calculations. The gas-phase adsorbate reference energies, *E*_*adsorbate*_(*r*_*adsorbate*_), for CO_2_ and H_2_O were determined from separate DFT relaxations.

Rerelaxing empty MOFs following MOF+adsorbate relaxation is a necessary step to avoid overestimating binding energies. This additional relaxation step is necessary when the adsorbate molecule breaks symmetry and allows the MOF to relax more fully compared to the relaxation of the empty MOF alone. In these examples, the new lower energy structure is a better representation of the true structure of the MOF than the structure taken from the CoRE MOF data set. The ODAC23 data set included hundreds of examples where rerelaxation of this kind was found to lead to lower energy empty MOF structures than the original DFT-relaxed structure.^28^ One possible origin of this outcome is that the original crystal structures were solved using simplifying symmetries that are not actually present in the lower energy structure. In these cases, the physically relevant adsorption energy would require using the lower-energy empty MOF system as a reference. This rerelaxation approach was not used systematically for all structures, however, in the ODAC23 study. This step was only performed in ODAC23 for a subset of materials due to the size of the data set, but the smaller scale of this work allows us to be rigorous for all 60 systems.

Single-point energies for the adsorbate molecules, *E*_*adsorbate*_(*r*_*sys*,*vacuum*_), were calculated in a 20 × 20 × 20 Å box to ensure that interactions with periodic images were negligible. Directly using MOF unit cells for adsorbate energy calculations can be problematic because MOF unit cells can be narrow in one direction. Self-interaction across periodic images (*E*_*adsorbate*,*self*_) can be non-negligible in such cases. Fortunately, the adsorbate self-interaction correction energies in this work were small because all of the MOF unit cells included in this study are sufficiently large. Thus, the *E*_*adsorbate*,*self*_ term in eq 6 was treated as negligible relative to the adsorption energies. It is common in HTS calculations to treat small adsorbate molecules as rigid, so we exclude *E*_*adsorbate*,*deform*_ from eq 6 and from all analysis. These assumptions are validated below. Since we seek to compare FFs and because we initialized each geometry using the same coordinates for each FF, neglecting these effects should have no effect on our qualitative conclusions.

Because any FF is not entirely consistent with DFT, computing FF adsorption energies of DFT-determined geometries that do not represent energy minima for the FFs is not practically relevant. To achieve a stringent test of the FFs and to emulate the approach commonly taken in existing literature, our FF energy calculations are all initialized using unrelaxed MOF geometries and adsorbate placements. Every FF energy presented in this work is determined from relaxations using the specified FF.

### DFT Parameters

DFT calculation parameters and initial adsorbate positions were taken directly from ODAC23, and full calculation details are available in that publication.^28^ Briefly, we used the PBE exchange-correlation functional^62^ with a D3 dispersion correction,^63^ a plane wave energy cutoff of 600 eV, and a 1 × 1 × 1 k-point grid. All calculations were spin-polarized and were performed in the Vienna Ab Initio Simulation Package (VASP) v5.^64^

We initialized empty MOF geometries here using the ODAC23 relaxed empty MOF geometries. Thus, most empty MOF relaxations converged quickly. A handful of MOF relaxations, however, did not converge quickly due to significant changes in the unit cell parameters relative to ODAC23. This occurred when empty MOFs were rerelaxed in the ODA23 work because the presence of a guest molecule in one of the ODAC MOF+adsorbate relaxations broke the initial MOF structure’s symmetry and yielded a lower-energy ground state empty MOF. The rerelaxations in ODAC23 for these examples did not relax unit cell parameters as we do here, so some differences arise between this work and the ODAC23 work for such MOFs.

### Classical Force Field Parameters

Our classical force field approach combined UFF4MOF^60,61^ for all MOF degrees of freedom with point charges from the density-derived electrostatic charges (DDEC) method^65^ taken directly from the ODAC23 work. Point charges on MOF atoms were only used in computing MOF-adsorbate interactions since UFF4MOF does not include charge contributions. TraPPE^66,67^ and the SPC/E model^68^ were used for CO_2_ and H_2_O, respectively. The SPC/E model was chosen over more complex water models such as TIP4P^69^ because of its simplicity. The ability to model water without placing massless sites was useful because the adsorbate configurations were taken directly from the ODAC23 work, which does not include massless sites.^70,71^ Lorentz–Berthelot mixing rules^72,73^ were used to define Lennard-Jones interactions. A cutoff of 12.5 Å without tail corrections was used for all UFF calculations, and long-range electrostatics were treated using an Ewald summation with a force accuracy of 10^–5^ kcal/mol/Å.^74^ While numerous other classical FFs have been used in HTSs to define MOF-adsorbate interactions,^23,75^ UFF4MOF is one of the few general-purpose classical FFs available to describe MOF degrees of freedom in flexible materials.^24,60,61^ The classical methods we selected here are expected to be largely representative of existing literature.

All classical FF calculations were performed in the Large-Scale Atomic/Molecular Massively Parallel Simulator (LAMMPS) package,^76^ with LAMMPS input files generated using lammps-interface.^23^ All LAMMPS input and log files are available in the SI. Unlike all other calculations in this work, we created supercells for UFF calculations such that the minimum supercell dimension was greater than double the 12.5 Å cutoff (>25 Å). This is required for LAMMPS to compute the energies of dihedral angles. Relaxations were performed using the Polak-Ribiere conjugate gradient algorithm^77^ with a force cutoff of 1.153 kcal/mol/Å (0.05 eV/Å) to match that from the DFT calculations. The maximum of the atomic forces was used to determine the force convergence rather than the default 2-norm of the global force vector, and a quadratic line search algorithm was used. For the empty MOFs, we performed 200 iterations of atomic coordinate relaxation followed by triclinic cell relaxation to avoid converging to local minima. The maximum volume change per iteration was set to 10% to aid convergence for one empty MOF relaxation exhibiting pressure instability (ID 9_66). MOF+adsorbate relaxations consisted of 10 iterations of geometry relaxation in a fixed unit cell. A maximum of 5,000 energy evaluations and 50,000 force evaluations per iteration were allowed for all calculations.

### ML Force Field Parameters

All ML force fields were used with default parameters. The M3GNet model was imported using the Materials Graph Library (MatGL) package with a default radial cutoff of 5 Å.^42^ CHGNet also used a cutoff of 5 Å, and the MACE models and eSEN used a radial cutoff of 6 Å. The EqV2-ODAC model used a radial cutoff of 8 Å and a maximum of 20 neighbors per atom.^28,78^ Unit cell relaxations for empty MOFs using MLFFs were performed using Python’s Atomic Simulation Environment (ASE)^79^ FrechetCellFilter. The empty MOF unit cell was not relaxed with EqV2-ODAC because the model does not predict stresses; the unit cell was also fixed for all MOF+adsorbate relaxations.

All MLFF relaxations were performed in ASE using the Broyden-Fletcher-Goldfarb-Shanno (BFGS) algorithm with a force tolerance of 0.05 eV/Å, a maximum atomic displacement per iteration of 0.05 Å, and a maximum of 1,000 iterations. As with DFT and UFF, MLFF relaxation energies for gas-phase CO_2_ and H_2_O were determined via geometry optimization in a 20 × 20 × 20 Å box with each respective MLFF. Although no MLFFs were trained with data from small molecules, our isolated molecule relaxations produced reasonable CO_2_ and H_2_O structures that were consistent with those from DFT. All Python scripts and log files are available in the SI.

### Initial Geometries for FF Relaxations

Since we are interested in the ability of FFs to self-consistently compute adsorption energies, we used unrelaxed CoRE MOF^80,81^ structures with coordinates *r*_*MOF*_^*CoRE*^ to initialize most empty MOF geometries. The CoRE superscript represents unrelaxed MOF coordinates taken directly from the CoRE MOF database.^80,81^ This was the same approach used in the ODAC23 work. In systems involving rerelaxed MOFs as described above for DFT calculations, we initialized MOF positions with the same positions used to initialize the ODAC23 rerelaxations. This ensures a fair comparison between our DFT and FF calculations; the systems affected by rerelaxation are noted by an asterisk in Table S1.

In all *E*_*ads*_, *E*_*int*_, and *E*_*static*_ calculations, initial adsorbate molecule placements were taken from ODAC23. Since relaxed unit cell parameters can differ between the FFs, the relative adsorbate molecule position was maintained to ensure a fair comparison between different models despite different FF-determined geometries for a given MOF. Specifically, the fractional coordinates of the molecule’s center of mass were retained, and the bond lengths and molecular orientation were identical to those in ODAC23. Adsorbate molecule positions were not adjusted for DFT calculations where the relaxed unit cell dimensions in this work closely matched those from ODAC23.

## Results

### Data Set Selection

The ODAC23 data set includes DFT data for more than 8,000 MOFs, including more than 3,400 structures containing point defects. Thoroughly implementing and testing UFF4MOF for a diverse range of structures is time-consuming when not assuming rigidity because LAMMPS structural file generation is nontrivial for large systems such as MOFs. Thus, we selected a relatively small subset of the ODAC23 materials for our calculations. This also reduced the overall computational cost.

A total of 60 MOF+adsorbate systems were taken from the ODAC23 in-domain “initial structure to relaxed energy” (IS2RE) test set for further study in this work. Only the test set was considered to avoid data leakage in the EqV2-ODAC predictions, and only systems with a single adsorbate molecule, either CO_2_ or H_2_O, were considered for simplicity. First, the MOFs in the IS2RE test set were screened for their DAC favorability according to ODAC23 DFT calculations. A MOF is considered favorable for DAC if, for all ODAC23 IS2RE single-adsorbate train, validation, and test MOF+adsorbate systems, the system with the most favorable DFT adsorption energy contains CO_2_ and not H_2_O. This is an unusual phenomenon within the complete spectrum of MOFs that exist, but it is highly desirable for DAC.^59^ Similarly, MOFs are also considered potentially favorable for DAC if the average adsorption energy for systems containing CO_2_ is more favorable (i.e., more negative) than that for systems containing H_2_O.

We used an algorithm to identify systems for this study, but some manual curation on a handful of systems was required to ensure a diverse and balanced data set of interesting materials. The algorithm used to select systems can be found in the SI. MOFs with an absolute DFT-calculated *E*_*MOF*,*deform*_ ≤ 0.05 eV per unit cell were considered to undergo negligible deformation, while MOFs with *E*_*MOF*,*deform*_ > 0.05 eV per unit cell were considered to undergo significant deformation. Systems were added to the data set to approximately balance the two deformation classes and adsorbate types while prioritizing DAC favorability and avoiding duplicates of MOFs.

One system (ID 3_78) was excluded from further study due to unit cell contraction and pore collapse upon DFT relaxation (see Figure S1), leaving a total of 59 systems in the data set. This material was rerelaxed with fixed unit cell parameters in ODAC23. When including cell parameters as degrees of freedom in this work, the resulting MOF geometry significantly differs from the geometry included in ODAC23. We calculated a positive DFT adsorption energy for CO_2_ in this material, making it unsuitable for adsorptive separations.

The ODAC23 data set includes information for large numbers of pristine MOFs and MOFs containing linker vacancies. Our data set here contains 31 systems derived from 28 pristine MOFs and 28 systems derived from 23 defective MOFs. About two-thirds of the systems contain MOFs that are potentially favorable for DAC according to our above definitions using the ODAC23 adsorption energies, with 20 very favorable systems, 19 potentially favorable systems, and 20 not favorable systems according to ODAC23 calculations. The systems not favorable for DAC were included because we could not identify 60 systems from the test set that satisfied the DAC favorability criteria. A summary of MOF+adsorbate systems we considered is given in Table S1.

Concerns regarding the validity of MOFs from computation-ready databases have recently arisen, and prior studies have suggested partial charge assignments can used as a surrogate for bond orders to screen MOFs for misbonded atoms.^82–84^ Several of these concerns have been addressed in the most recent version of the CoRE MOF database.^85^ Jin et al. reported in their recent comment on ODAC23 ^86^ that around 40% of the CoRE MOFs used in ODAC23 where “charged” according to MOFChecker v2,^87^ which uses a combination of coordination checks and predicted metal oxidation states from the OxiMachine ML algorithm.^88^

Jin et al. flagged as “charged” 13 of the 46 pristine CoRE MOFs from which the MOFs used in this study are derived. We used the MOFChecker v0.9.6 to confirm that partial charge assignments from the EQeq method^89^ are physically reasonable in every system we examined. For additional study, DDEC charges derived from the ODAC23 DFT calculations are also available in that database. DDEC charges are expected to be more accurate than the charge equilibration methods used in other publications,^65^ and examples are known where DDEC charges derived from DFT calculations are physically reasonable even when EQeq charges are not.^90^ Table S2 lists the 13 pristine CoRE MOFs flagged by Jin et al. and provides both EQeq and DDEC charges for every atom in one such MOF (ESURER03). The DDEC charges are reasonable and match those from EQeq reasonably well despite the MOF being flagged as ”charged” with a net MOFChecker charge of +4. Since we are focused on studying FF performance instead of particular promising MOFs and are using converged DFT calculations in neutral cells as benchmarks, we acknowledge but do not dwell on this topic of ongoing debate.

### DFT Calculations in Rigid and Deformable MOFs

We first consider the results of our DFT calculations separate from the FFs. Figure 2(a) shows an overview of our DFT *E*_*ads*_ and *E*_*int*_ split into negligible deformation (blue) and significant deformation (red) classes. Markers are colored with darker and lighter shades to denote the presence of a CO_2_ or H_2_O guest molecule, respectively. According to eq 6, larger deviations from the parity line indicate larger MOF deformation energies. Our data set also represents a mix of physisorption and chemisorption with 44 and 15 systems, respectively, where chemisorption is defined as a DFT *E*_*int*_ < −0.5 eV. We use *E*_*int*_ to define chemisorption since it directly probes the contribution of electronic interactions that underlie the covalent bonding characteristic of chemisorption. *E*_*ads*_ remains the most relevant quantity in this work, but MOF deformation can weaken *E*_*ads*_ even when chemisorption is occurring as indicated by *E*_*int*_.

**Figure 2:**
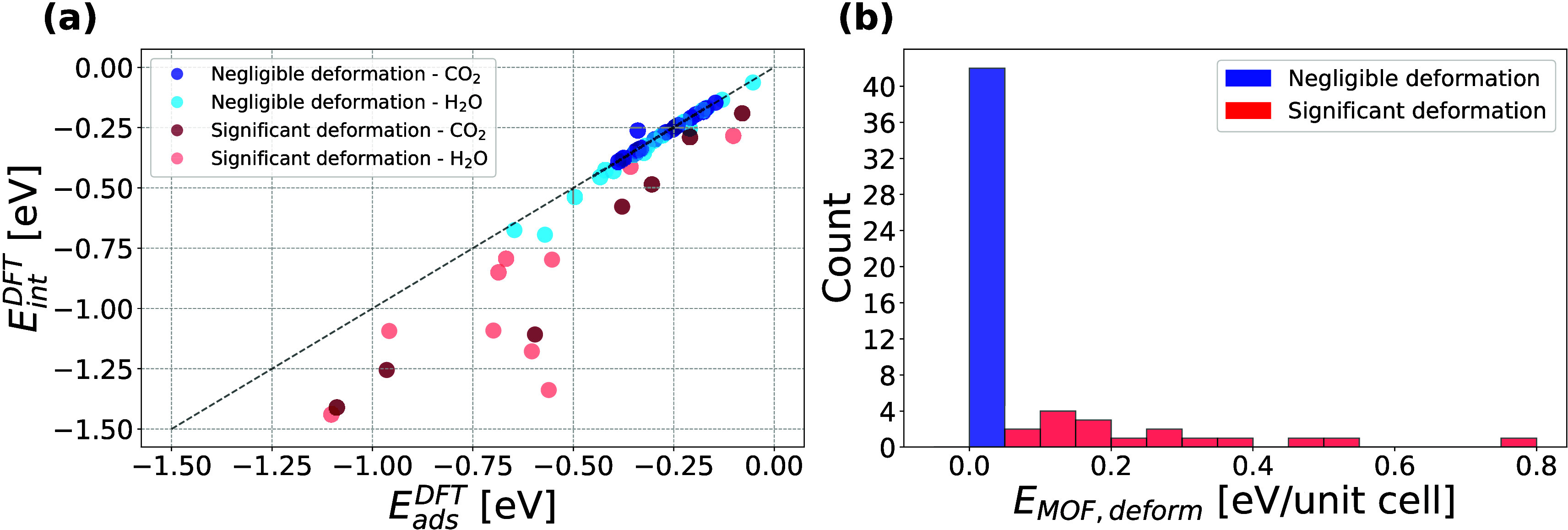
(a) Distribution of DFT adsorption and interaction energies in the 59 selected MOF + adsorbate systems. (b) Histogram of MOF deformation energies colored by deformation class.

Figure 2(b) shows the corresponding MOF deformation energies. Positive deformation energies indicate that relaxation in the presence of the adsorbate molecule yields a MOF structure that is less energetically favorable than the empty MOF relaxed alone (as in Figure 1). Upon removal of the adsorbate molecule, these MOFs return to their empty ground state. The one point noticeably above the parity line in Figure 2(a) is system 1_230, which has a *E*_*MOF*,*deform*_ of 0.002 eV but lies above the parity line due to its attractive *E*_*adsorbate*,*self*_ of –0.08 eV.

The 59 systems are not split evenly between the negligible and significant deformation classes because our DFT calculations include some differences from those from ODAC23 on which our materials selections were based. Rerelaxation of empty MOFs after removing the guest molecule led to multiple examples where MOF deformation would have been classified as significant using ODAC23 data but were reclassified as negligible MOF deformation cases once lower energy empty MOF structures were found in our current calculations. Of the 42 systems with negligible deformation, 22 (20) contain CO_2_ (H_2_O), and of the 17 systems with significant deformation, 7 (10) contain CO_2_ (H_2_O).

The “Deformation class” column in Table S1 shows whether our DFT calculation for each system indicated physisorption or chemisorption of the guest molecule. Whether physi- or chemisorption occurs is a function of both the MOF itself (e.g., OMSs) and our specific adsorbate placement. Unsurprisingly, most significant MOF deformations occurred in systems where the adsorbate molecule chemisorbed since the adsorbate more closely interacts with framework atoms in these cases.

We found four MOFs (system IDs 3_384, 6_73, 6_239, 9_289) that deformed more than the 0.05 eV/unit cell cutoff despite physisorbing the adsorbate molecule. Several factors may contribute to this surprising result. First, some of the MOF deformation energies are only slightly larger in magnitude than the cutoff, including systems 3_384 (0.050 eV/unit cell) and 9_289 (0.111 eV/unit cell). All of the MOF deformation energies in significantly deforming MOFs featuring physisorption are less than 0.2 eV/unit cell, which is much smaller than the largest deformation of 0.757 eV/unit cell. Second, MOFs with many atoms in the unit cell will have larger deformation energies because deformation typically manifests as small atomic position changes across many atoms, as shown in Figure 1. System 3_384 has more atoms in its unit cell than any other MOF in this study at 495. Systems 6_73, 6_289, and 9_289 have 177, 257, and 259 atoms/unit cell, respectively, placing all four in the top half of MOFs ranked by atom count. Lastly, the distance from the MOF and orientation of the adsorbate can affect the magnitude of deformation. In system 3_384, for example, the distance between the guest H_2_O and the nearest MOF atom is less than 2 Å. Figure S2 shows the placement of CO_2_ in systems 6_73 and 4_194, where the guest orients parallel to and semiorthogonal to the pore’s surface, respectively. The shortest distance between the CO_2_ and the nearest MOF atom is 2.6 Å in both systems. The parallel orientation causes a greater MOF deformation compared to an orthogonal orientation because all three atoms in the adsorbate are interacting strongly with the MOF atoms. A combination of these factors helps explain why physisorption may result in significant MOF deformation.

#### Adsorption in Rigid MOFs

It is also interesting to consider whether MOF deformation makes a meaningful difference in adsorption energies for simulations commonly performed in the literature, namely GCMC simulations with rigid MOFs. Figure 3 compares *E*_*ads*_ calculations in deformable MOFs to *E*_*static*_ using DFT. The result for system 0_217, the example shown in Figure 1, is highlighted in green.

**Figure 3:**
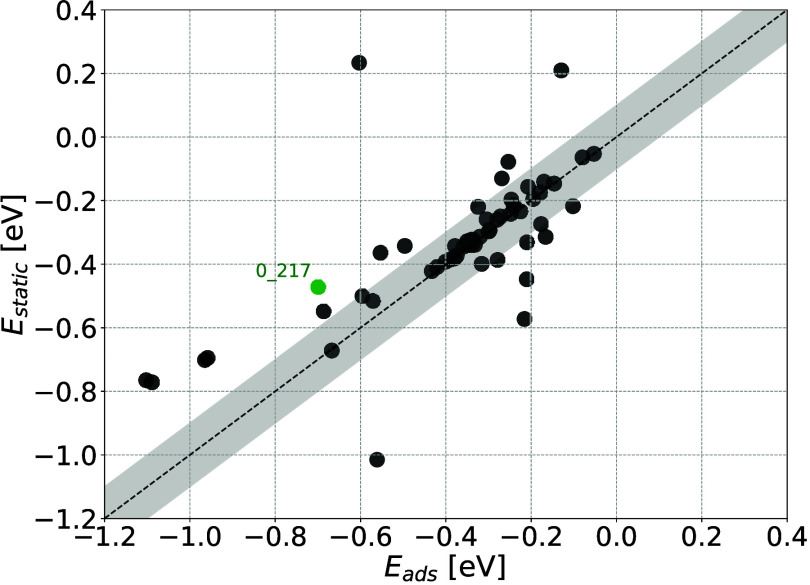
Comparison of *E*_*ads*_ with fully deformable MOFs to *E*_*static*_ in rigid MOFs using DFT. System 0_217 from Figure 1 is highlighted in green. The shaded area within ± 0.1 eV indicates the region in which good agreement between *E*_*ads*_ and *E*_*static*_ is achieved.

Systems that do not significantly deform naturally fall close to the parity line in Figure 3. The deviation between *E*_*static*_ and *E*_*ads*_ is considerable, particularly in the chemisorption regime from −1.2 eV < *E*_*int*_ < −0.5 eV where most chemistries promising for DAC occur. Nearly 30% of all tested systems fall more than 0.1 eV from the parity line. Including all 59 systems, the MAE between these two quantities is 0.268 eV when −1.2 < *E*_*int*_ < −0.5 and 0.056 eV when −0.5 ≤ *E*_*int*_ < 0.0 eV. These results indicate that neglecting adsorbate–induced MOF deformation can lead to significant errors in determining the adsorption affinity of molecules.

Figure S3 shows that there is also no correlation between the suitability of the rigidity assumption and the magnitude of MOF deformation and pore limiting diameter (PLD) of the relaxed empty MOF. That is, it is not straightforward to know *a priori* for which MOFs the rigidity assumption is reasonable. These results hint that many of the materials of greatest interest for DAC cannot readily be modeled using rigid MOF structures.

#### Validation of Adsorbate Assumptions

We conclude our study of the DFT results by validating our assumptions regarding adsorbate self-interaction (*E*_*adsorbate*,*self*_) and deformation (*E*_*adsorbate*,*deform*_). In our calculations only two DFT adsorbate self-interaction energies exceeded 0.01 eV with a maximum of 0.079 eV. Thus, we neglect *E*_*adsorbate*,*self*_ from eq 6 in our analysis. Likewise, the maximum and average DFT *E*_*adsorbate*,*deform*_ in our data set are 0.078 and 0.0025 eV, respectively. The largest adsorbate deformation energies represent only minor changes in the geometry of the adsorbate molecule (e.g., O–C–O angle). In the case with the largest adsorbate deformation energy of 0.078 eV, the corresponding MOF deformation energy is 0.50 eV, meaning that only 13% of the total deformation energy is due to the adsorbate. In total, 50 of 59 systems have an absolute DFT adsorbate deformation energy of less than 0.01 eV. For these reasons, we lump *E*_*adsorbate*,*deform*_ in eq 6 into the *E*_*MOF*,*deform*_ term with the understanding that MOFs contribute almost all of the deformation energy for a given system.

### FF Calculations in Deformable MOFs

Errors in FF *E*_*ads*_ calculations relative to DFT can arise because of errors in FF descriptions of MOF–adsorbate interactions (*E*_*int*_) or because the FF treats the MOF as either too rigid or too deformable (*E*_*MOF*,*deform*_). In this section, we decompose FF *E*_*ads*_ predictions as outlined in eq 6 to investigate the relative magnitudes of these effects. DFT energies are taken as the ground truth for error calculations.

#### Classical Force Field (UFF)

The mean absolute errors (MAEs) in adsorption energies using UFF for all 59 systems are shown in Table 2 and Figure 4. UFF is better at describing MOFs with negligible deformation energies then MOFs with significant deformation energies. Table 2 shows that UFF struggles to describe chemisorption, defined here as DFT *E*_*int*_ < −0.5 eV. This is unsurprising because UFF is not a reactive FF. We will see below, however, that UFF approaches the adsorption energy MAE of the best performing MLFFs when only considering physisorption. Figure 3, however, reminds us that the rigidity assumption is frequently invalid and that UFF cannot be used in a simple way to detect this outcome.

**Table 2:** Mean Absolute Errors in eV Relative to DFT Split between Physisorption and Chemisorption for Each Energy Term Using Each FF in This Study^a^

force field	subset	*E*_ads_	*E*_int_	*E*_MOF,deform_
UFF	Physisorption	0.116	0.109	0.026
	Chemisorption	0.391	0.618	0.217
	Total	0.186	0.238	0.074
M3GNet	Physisorption	0.136	0.128	0.020
	Chemisorption	0.575	0.799	0.205
	Total	0.247	0.298	0.067
CHGNet	Physisorption	0.092	0.095	0.018
	Chemisorption	0.217	0.362	0.202
	Total	0.124	0.163	0.065
MACE-MP-0	Physisorption	0.161	0.121	0.057
	Chemisorption	0.238	0.364	0.210
	Total	0.181	0.183	0.096
MACE-MPA-0	Physisorption	0.365	0.366	0.023
	Chemisorption	0.334	0.288	0.149
	Total	0.357	0.346	0.055
eSEN	Physisorption	0.187	0.192	0.019
	Chemisorption	0.409	0.637	0.221
	Total	0.243	0.305	0.070
EqV2-ODAC	Physisorption	0.182		
	Chemisorption	0.220		
	Total	0.191		

a There are 44 and 15 systems in the physisorption and chemisorption sets, respectively.

**Figure 4:**
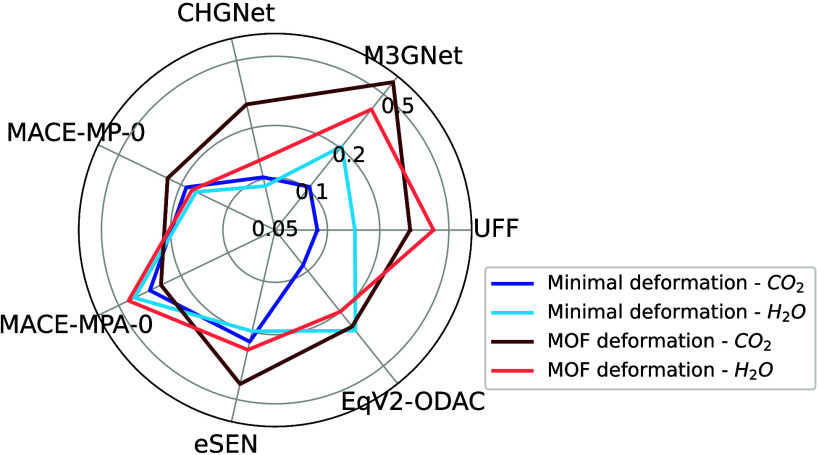
Mean absolute adsorption energy errors relative to DFT in eV separated by deformation class for FF relaxations. The radial axes show energies on a logarithmic scale with the origin representing a MAE of 0.05 eV.

We reiterate that every FF adsorption energy calculation was performed self-consistently, so the empty MOFs used in the FF relaxations differ from those used in DFT. This stringent test of the FFs is intended to mimic a standard FF adsorption energy workflow without bias from DFT calculations. A comparison of UFF adsorption energy predictions to DFT in only DFT-relaxed MOFs is shown in the ODAC23 work, and we found very good agreement between the two methods in the physisorption regime.^28^

The decomposition of adsorption energy into interaction and deformation energies in eq 6 presents a useful way to further explore the cause of adsorption energy errors. A low adsorption energy error may be the result of cancellation of errors between *E*_*int*_ and *E*_*MOF*,*deform*_, so it is not enough to only consider adsorption energy when evaluating the accuracy of FFs. FFs suitable for predicting adsorption behavior in nonrigid MOFs must adequately describe both MOF–adsorbate interactions and MOF deformation. Figure 5 shows signed adsorption energy errors labeled by system ID and separated by adsorbate with the corresponding interaction and MOF deformation energy errors. Only systems undergoing significant deformation are shown in Figure 5; the same plot for systems that undergo negligible deformation is shown in Figure S4. The coloring of Figures 5 and S4 shows larger errors relative to DFT as darker shades of pink (negative) and green (positive). Cancellation of errors occurs when the right triangle (*E*_*MOF*,*deform*_) and the bottom triangle (*E*_*int*_) differ in color, signifying a positive error relative to DFT for one quantity and a negative error for the other. The resulting adsorption energy error is then lower than the maximum of the interaction and MOF deformation energy errors. An example of this is system 2_74 for all five FFs.

**Figure 5:**
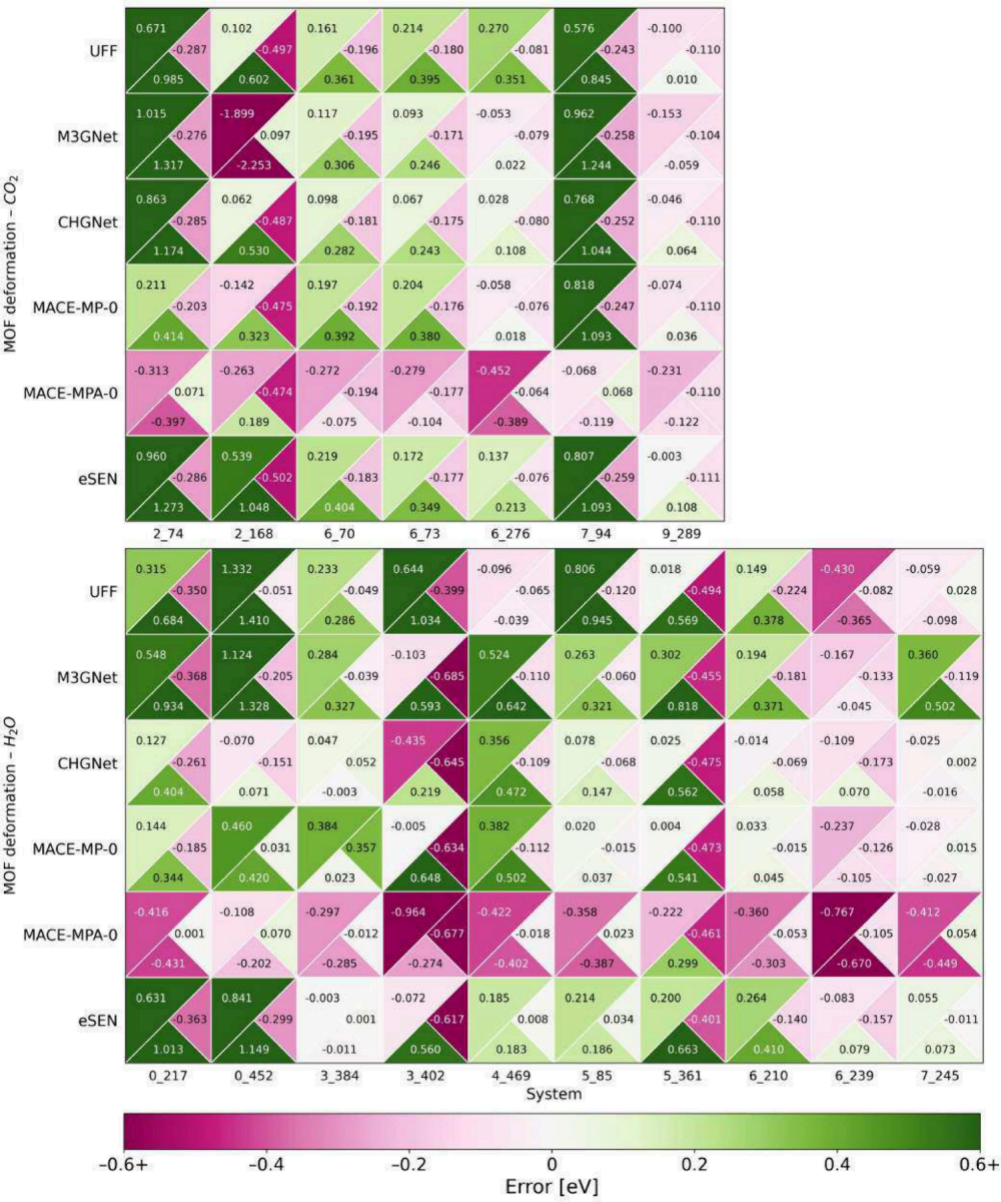
Adsorption energy errors (upper left triangle), interaction energy errors (bottom triangle), and MOF deformation errors (right triangle) from FF relaxations for systems that undergo significant deformation separated by adsorbate. All energies are in eV using the color bar shown in the figure.

UFF errors are shown on the first row in Figures 5 and S4. Interaction energy errors are consistently large, indicating that UFF fails to adequately describe nonbonded interactions and, in the case of chemisorption, chemical bonding between the host and guest molecules. UFF also performs poorly when predicting MOF deformation energies for MOFs that significantly deform according to DFT, as indicated by the darker shades of color in the right triangles. Overall, there is little discernible pattern across the UFF errors, which is consistent with the idea that the classical FF is sensitive to slight changes in atomic positions and electrostatics. The UFF adsorption energy MAE of 0.186 eV is substantially greater than that of the best-performing MLFF.

The top row of Figures 5 and S4 also shows that cancellation of errors is common in our UFF calculations, occurring in 42 of 59 systems. Significant cancellation of errors in nearly half of systems, meaning that UFF often performs more poorly describing interaction energy or MOF deformation energy than it does describing the overall adsorption energy.

#### Foundational MLFFs

The MAEs for the four foundational MLFFs – M3GNet, CHGNet, both MACE models, and eSEN – are shown in Table 2 and Figure 4. The *E*_*ads*_ MAEs for M3GNet, CHGNet, and MACE-MP-0 outperform that of UFF, but MACE-MPA-0 severely underperforms and exhibits the largest MAE of any FF tested. CHGNet and MACE-MP-0 perform best of all total energy models tested with moderate to significant improvements in *E*_*int*_ and *E*_*MOF*,*deform*_ accuracy relative to both UFF and M3GNet. Like all the MLFFs, CHGNet and MACE-MP-0 struggle with chemisorption compared to physisorption, but the performance disparities between the two adsorption types are lower than for other FFs.

It is not surprising some MLFFs outperform UFF because they were trained on databases of DFT calculations. Even still, percent errors in adsorption energy approach and, in some cases, exceed 100%. To determine whether these errors are reasonable, consider the published test set errors for CHGNet and for MACE-MP-0. CHGNet achieves an MAE of 33 meV/atom on its MPtrj test set,^43^ and MACE-MP-0 achieves an MAE of 33 meV/atom for MOF total energy predictions.^46^ The MOFs in our data set range from 66 to 492 atoms/unit cell with an average of 187, corresponding to average total energy errors from 2.2 to 16 eV per unit cell with both CHGNet and MACE-MP-0. While much of these errors may cancel during an adsorption energy calculation, it is not unreasonable to find resulting *E*_*ads*_ errors on the same magnitude of *E*_*ads*_ itself. That is, the good performance of MLFFs on their test sets does not necessarily translate to low percent errors in adsorption energy.

The relatively poor performances of MACE-MPA-0 and eSEN given their sizes are unexpected and should be further investigated, but why MLFFs perform well or poorly is beyond the scope of the current work. Both FFs contain more parameters and were trained on more structures than the other models considered in this work.

The decomposition of errors is shown in rows 2–6 of each block in Figures 5 and S4. Cancellation of errors occurs in 33 systems for M3GNet, 37 systems for CHGNet, 28 systems for MACE-MP-0, 39 systems for MACE-MPA, and 33 systems for eSEN. The ability of foundational MLFFs to describe van der Waals interactions with reasonable accuracy is particularly interesting and is worthy of further study given the lack of training data that includes dispersion effects in the MP database. Despite improvement over UFF, *E*_*ads*_ errors remain significantly greater than the 0.1 eV target discussed above for all MLFFs, meaning no FF is yet accurate enough for reliably studying adsorption in deformable MOFs.

#### ODAC23 Equiformer (EqV2-ODAC)

Table 2 and Figure 4 show that EqV2-ODAC performed similarly to both CHGNet and MACE-MP-0. EqV2-ODAC is excluded from some columns of Table 2 and from Figures 5 and S4 because the model directly predicts adsorption energy and therefore cannot decompose errors into interaction and MOF deformation contributions. We conclude that EqV2-ODAC successfully learned the error cancellation implicitly from DFT. It is also interesting that the top-performing MLFFs rival EqV2-ODAC given the latter’s underlying training data exclusively containing CO_2_ and H_2_O adsorption in MOFs and its more than 153 million parameters.

## Discussion

### Deformation as a Classification Problem

We have established that existing MLFFs can outperform a classical FF for describing adsorption in deformable MOFs in terms of MAE, although neither approach has sufficient accuracy for detailed calculations. It is also useful to address whether MLFFs can outperform classical FFs in determining whether a MOF will undergo significant deformation upon the introduction of a guest molecule. We used a threshold of 0.05 eV to classify systems as having significant MOF deformation or not. The classification is performed implicitly based on the FF results, and no explicit classification model is used. As above, DFT results are taken as the ground truth for this analysis.

Figure 6 shows the confusion matrices for this classification task based on relaxation calculations for each FF. EqV2-ODAC was again omitted because it cannot decompose *E*_*ads*_. All six FFs notably performed very similarly, and there is not one clear best model based solely on this analysis. CHGNet and MACE-MPA-0 both misclassified only 17% of the data set despite MACE-MPA-0 performing worst in terms of *E*_*ads*_ MAE. This is consistent with most MACE-MPA-0 energy errors resulting from the *E*_*int*_ term in eq 6. When only considering MOFs that undergo significant deformation, all FFs except MACE-MP-0 and MACE-MPA-0 misclassify more than half of the systems as undergoing negligible deformation. That is, the FFs tend to underpredict MOF deformation where DFT indicates the phenomenon is important. These findings indicate that energy MAEs are not necessarily correlated to whether an FF is able to identify which systems undergo significant MOF deformation. Although MLFFs can outperform UFF in terms of energy MAE, more work is needed to tailor FFs that can adequately describe adsorbate-induced MOF deformation and that can readily identify systems in which such deformation is significant.

**Figure 6:**
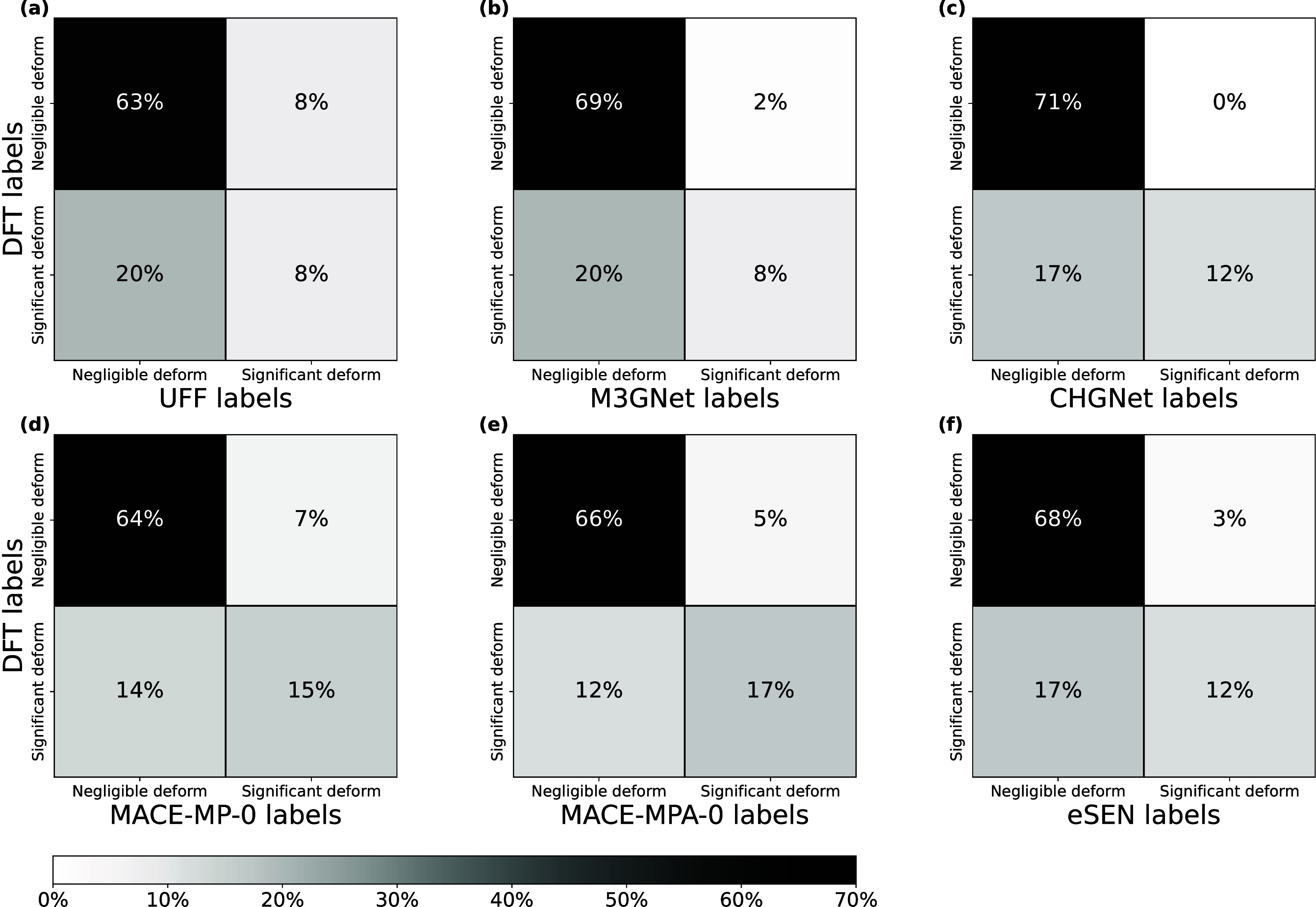
Confusion matrices for deformation class identification for (a) UFF, (b) M3GNet, (c) CHGNet, (d) MACE-MP-0, (e) MACE-MPA-0, and (f) eSEN. True positives and true negatives are located in the upper left and lower right of each matrix, respectively.

A natural question regarding these results is whether large errors were associated with specific systems that were challenging to model. Figure S5 shows that systems with outlier errors for one model rarely yielded large errors for other models. That is, the errors were not the result of specific geometries across the four FFs tested. Figure S6 shows unit cell volume errors from FF relaxations relative to those from DFT. These results were consistent with the energy findings presented here, with the MLFFs generally describing unit cell geometry changes better than UFF.

### Computational Cost Comparison

The primary advantage of FFs is their greatly reduced computational cost relative to DFT. Table S3 provides the cost in CPU hours of running the entire set of calculations presented in this work for each FF. CHGNet, the fastest FF, represents a cost savings of 4 orders of magnitude, whereas eSEN, the slowest FF, still represents a savings of 2 orders of magnitude. We do not propose strategies for designing faster MLFFs but instead provide this comparison to aid readers in selecting general-purpose MLFFs for their future work. The UFF cost is higher than expected in Table S3 because we used supercells for UFF but not for any other model. The UFF compute time is 3.2 h, the lowest of any FF, when considering calculations using unit cells. The costs of each model roughly correspond to their sizes as outlined in Table 1. EqV2-ODAC is a notable exception to this trend since its reduced computational cost relative to MACE-MPA-0 and eSEN is due to being a direct adsorption energy FF that thus requires only one relaxation to compute *E*_*ads*_.

### Limitations of FF Energy Predictions

It is of course interesting to ask whether any of the FFs we tested accurately describes the systems we considered. One limitation of the current study is the relatively small sample size compared to the size of typical MOF databases. Given the relatively small differences in the performance of the FFs tested, the sample size of 60 is likely not sufficient to draw a strong conclusion about which FF is best for MOF deformation. However, the sample size is sufficient to compute meaningful statistics about the overall performance of the models. Table S4 reports the *R* ^2^ and Pearson correlation coefficient values for CO_2_ and H_2_O adsorption energies in the deformable MOFs in our data set. The definition of *R* ^2^ used in this work is

R2=1−∑i(yi−yi^)2∑i(yi−y̅)2 8

where *y*, *ŷ*, and *y̅* are the true target values, FF predicted values, and mean of the true target values, respectively. *R* ^2^ can be negative if the FF predictions are worse than predicting the mean of the DFT *E*_*ads*_. Despite promising MAEs for the top performing FFs, only modest correlation exists between the DFT and FF results, even for the best performing FF. Figure S7 visually confirms this poor correlation for all FFs, especially in the physisorption regime. This poor performance points to complexity of the task, which requires an accurate treatment of both adsorption and chemical bonding within the MOF framework. The magnitude of typical adsorption energies (∼0.4 eV) and deformation energies (∼0.1 eV) are similar to the magnitude of the *E*_*ads*_ MAEs of the FFs (∼0.2 eV), making it unsurprising that correlations are often poor. The poor results for *R* ^2^ may be biased by the fact that the examples chosen were selected to over-represent deformation and chemisorption. However, deformation and chemisorption are particularly relevant for DAC, and our results suggest that currently available FFs are not robustly capable of describing these phenomena.

A particularly challenging aspect of using FFs to compute adsorption energies is distinguishing between physisorption and chemisorption. The results include several systems where DFT indicates that chemisorption is occurring but where FFs relax to local minima corresponding to physisorption. Table S5 highlights three systems where DFT and FFs predict chemisorption and physisorption, respectively. The distances between the adsorbate and MOF in the DFT-relaxed geometries are less than 2 Å, consistent with the strong DFT adsorption energies. The errors are consistently high due to FFs predicting physisorption, which skews the *R* ^2^ metric. Physisorption energies have smaller magnitudes and variances (–0.5 ≤ *E*_*ads*_ < 0 eV) compared to chemisorption, which has a higher magnitude and variance (–1.2 ≤ *E*_*ads*_ < –0.5 eV). Physisorption is also more prevalent in both ODAC and this work, with 85% of ODAC23 systems with one guest molecule and with 75% of the systems in this work having *E*_*ads*_ > −0.5 eV. Thus, it is possible for MLFFs to achieve a low MAE by fortuitously guessing physisorption energies. This is supported by the fact that M3GNet, CHGNet, MACE-MP-0, and eSEN all show low MAEs for physisorption despite having no D3 corrections included in their training data. Their strong performance is not necessarily an indication that they are correctly capturing the underlying physics. These findings further illustrate that MAE is not a good metric for assessing the ability of models to predict chemisorption.

Another known challenge associated with with adsorption energy predictions is that they require an accurate reference energy for the gas-phase adsorbate molecule (*E*_*adsorbate*_), which can be challenging for MLFFs to compute given their underlying training data. None of the training data for the four general-purpose MLFFs contains gas-phase molecules, though MP does contain CO_2_ and H_2_O in a variety of bulk configurations.^48^ In this work, we relied on the FFs to compute *E*_*adsorabte*_ despite their known limitations. An alternative approach that improves accuracy is to treat *E*_*adsorbate*_ as a free variable when computing the adsorption energies and to regress them to known targets. We determined a correction for the CO_2_ and H_2_O gas-phase energies for each FF using linear regression in scikit-learn^91^ to remove systematic errors between FF and DFT predictions (see eq 9 in the SI for details). Table S6 compares MLFF performance using direct MLFF *E*_*adsorbate*_ predictions and using this correction scheme. UFF is shown as a reference despite not suffering from the same challenges in principle. While the MAEs decrease, the ranking of the MLFFs remains similar, except for MACE-MPA-0, which becomes the second most accurate FF after correcting *E*_*adsorbate*_. This suggests that systematic errors in calculating gas-phase molecular energies can have an impact on the overall accuracy of some models, but they do not affect the conclusions of this work since the rankings of the FFs based on MAE and *R* ^2^ do not significantly change.

## Conclusions

Adsorbate-induced deformation is essential for accurately describing and understanding the mechanisms of adsorption in MOFs.^18,24,27^ We have developed a mathematical framework for probing the effects of adsorbate-induced MOF deformation, and we have leveraged Open DAC 2023 to show that such deformation can play a significant role in computing DFT adsorption energies in MOFs potentially favorable for DAC. We then explored the efficacy of a classical FF model and six MLFFs for describing MOF deformation induced by CO_2_ and H_2_O adsorption. Our results show that UFF – the most common FF used in previous MOF studies – is a poor emulator of DFT and does not accurately model chemisorption and MOF deformation in most cases. MLFFs trained on large databases of DFT calculations such as CHGNet and MACE-MP-0 performed better than UFF. Other MLFFs – namely M3GNet, MACE-MPA-0, and eSEN – performed similarly to or worse than UFF. More work needs to be done to determine why MACE-MPA-0 performs poorly for describing adsorption in MOFs, and we hypothesize this may be related to the model’s underlying data or size. The EqV2-ODAC model trained on the Open DAC 2023 work^28^ also performed well for predicting adsorption energies, which is not surprising given its underlying training data and size. However, these models all performed poorly when treating MOF deformation as a classification problem and still produce mean absolute adsorption energy errors relative to DFT significantly greater than a 0.1 eV target.

We intend for this work to serve as a useful starting point and motivator for future work focused on developing MLFFs for MOF applications. We have performed the calculations here to be similar to what is currently done in the literature so as to accurately represent the way that most MOF modelers use these models in their own work. Adsorbate-induced deformation must be adequately described by any ML model seeking to emulate ab initio method performance at a reduced computational cost. Pretrained MLFFs have improved rapidly for describing a wide range of materials, including MOFs, though the particular ML features underlying this success remain unclear. Despite this recent progress, however, work is still needed to develop MLFFs that can reliably describe the underlying physics of adsorption in solid sorbents relevant for DAC.

## Supplementary Material



## Data Availability

Data is available free of charge at https://zenodo.org/records/16848429.

## Notes

Algorithms for MOF selection and DAC favorability; description of selected adsorption systems; comparison of EQeq and DDEC charges for MOF ESURER03; additional MOF visualizations; adsorption energy decompositions for negligibly deforming MOFs; error correlations across all FFs; comparison of FF-relaxed unit cell volumes; computational cost comparisons; correlation coefficients with and without gas-phase adsorbate energy corrections; parity plot of all FF and DFT adsorption energies; relationship between FF errors and DFT-relaxed MOF+adsorbate geometries; MOF and adsorbate structural files; DFT and FF inputs and outputs; example MLFF relaxation scripts (PDF)
