# Supplementary material for: Comparing Classical and Machine Learning Force Fields for Modeling Deformation of Metal–Organic Frameworks Relevant for Direct Air Capture
Source: J Phys Chem C Nanomater Interfaces. 2025 Sep 8;129(37):16811–25. doi: 10.1021/acs.jpcc.5c04020 (PMC12451742; doi:10.1021/acs.jpcc.5c04020)
Supplement: Supplementary file 1 [file jp5c04020_si_001.pdf]

Supplementary Information for "Comparing Classical and Machine Learning Force Fields for Modeling Deformation of Metal–Organic Frameworks Relevant for Direct Air Capture"

Logan M. Brabson,[†] Andrew J. Medford,[*,†] and David S. Sholl[*,‡]

†*School of Chemical and Biomolecular Engineering, Georgia Institute of Technology, Atlanta, Georgia 30332, United States*

‡*Oak Ridge National Laboratory, Oak Ridge, Tennessee 37830, United States*

E-mail: ajm@gatech.edu; shollds@ornl.gov

Data Availability Statement The full ODAC23 dataset and all the trained ML models are publicly available at the official ODAC website (`https://open-dac.github.io/`) and the open-source GitHub repository (`https://github.com/facebookresearch/fairchem/tree/main`). All scripts, input, and output files for the calculations presented in this work are available in the supplementary `deform_public.zip` file. `README.md` in `deform_public.zip` contains a detailed file structure and description for each directory and file type. An outline of the directory is provided below.

1. `analysis` subdirectory:
   (a) `image_files`: Image files for each figure in .png format
   (b) `analysis.ipynb`: Python code for figure generation
   (c) `deformation_figures_data.xlsx`: Data for each figure in spreadsheet format
   (d) `deform_dataset_w_{FF}_final.json`: Raw data file for each force field tested
2. `build_dataset` subdirectory:
   (a) `build_dataset.py`: Python script for selecting MOFs from ODAC23 for use in this study
   (b) `dataset_IDs`: List of IDs output by `build_dataset.py` split by type of deformation indicated by ODAC23 DFT
   (c) `get_mof_favor.py`: Python script for evaluating a MOF's favorability for DAC based on ODAC23 DFT
   (d) `mof_favor.out`: Lists of MOFs classified as favorable (F), potentially favorable (PF), and not favorable (NF) for DAC
3. `example_mlff_workflow` subdirectory: Python scripts for M3GNet relaxations, which were used for all MLFF relaxations by changing the ASE calculator
   (a) `setup_empty_mofs.py`: Import empty MOF geometries from CoRE MOF and initialize with DDEC charges
   (b) `empty_relax.py`: Relax empty MOF atomic positions + unit cell
   (c) `combo_setup.py`: Place adsorbate molecules in relaxed MOFs
   (d) `combo_relax.py`: Relax combined MOF + adsorbate configurations, then perform single point calculation of the resulting empty MOF and adsorbate geometries
   (e) `frame_relax.py`: Re-relax the empty MOF output by `combo_relax.py`
   (f) `rigid_setup.py`: Place adsorbate in most energetically favorable empty MOF
   (g) `rigid_relax.py`: Relax combined MOF + adsorbate configuration while holding MOF positions fixed
   (h) `ads_relax.py`: Relax gas-phase (vacuum) CO_2 and H_2O molecules
   (i) `compile_energies.py`: Compute derived properties (e.g., E_ads, E_int, E_mof,deform) and write to file
   (j) `multi_regress_check.py`: Compute errors when considering gas phase adsorbate energy to be a free variable (see Table S4)
4. `outputs` subdirectory: Input and relaxed structures in CIF format, total energies used for adsorption energy calculations

(a) DFT: VASP inputs and outputs

(b) UFF: LAMMPS inputs and outputs

(c) M3GNet

(d) CHGNet

(e) MACE (MP-0 and MPA-0)

(f) eSEN

(g) EqV2–ODAC

Algorithm 1 Dataset selection algorithm

```
while len(dataset) < 60 do
    for each sys in IS2RE test set do
        ads ← get_ads(sys)
        favor ← get_DAC_favor(sys)
        if |E_MOF_deform| < 0.05 eV then
            class ← "Negligible Deformation"
        else if |E_MOF_deform| ≥ 0.05 eV then
            class ← "Significant Deformation"
        else
            skip

        if MOF not in class dataset then
            if len(class dataset) < 30 then
                if len(sys with ads in class dataset) < 15 then
                    if favor is very then
                        dataset.append(sys)
                    else if favor is potential and no remaining very favorable systems then
                        dataset.append(sys)
                    else if no remaining potentially favorable systems then
                        dataset.append(sys)
```

Table S1: Summary of MOF+adsorbate systems considered in this study

ID	MOF (CSD)	MOF (common name)	Defective	Adsorbate	Adsorption type	Deformation class	DAC favorability
0_72*	QOPCEE_0.06_0		Yes	CO_2	Physisorption	Negligible	No
0_227	CAYDIR		No	CO_2	Physisorption	Negligible	No
1_230	DAWBUA		No	CO_2	Physisorption	Negligible	Potential
1_277	WORSUT		No	CO_2	Physisorption	Negligible	Potential
2_45	JOHSIJ_0.07_1		Yes	CO_2	Physisorption	Negligible	Yes
2_173	ESURER03		No	CO_2	Physisorption	Negligible	No
3_74	BETGUE_0.12_0		Yes	CO_2	Physisorption	Negligible	Potential
3_88	IRELAU_0.12_0		Yes	CO_2	Physisorption	Negligible	Potential
3_144	UTEWOG_0.12_0		Yes	CO_2	Physisorption	Negligible	Yes
4_194*	FARBIL	MOF-48	No	CO_2	Physisorption	Negligible	Potential
4_207	JIZJOT		No	CO_2	Physisorption	Negligible	Yes
5_100	EGEJIK		No	CO_2	Physisorption	Negligible	Potential
5_134	PIWSEV		No	CO_2	Physisorption	Negligible	Yes
5_145	SIVWUR		No	CO_2	Physisorption	Negligible	Yes
5_186	PIWSEV		No	CO_2	Physisorption	Negligible	Yes
6_97	QUSSII_0.08_0		Yes	CO_2	Physisorption	Negligible	Potential
7_91	MIMFOF_0.03_1	AIPO-BDA	Yes	CO_2	Physisorption	Negligible	Yes
7_112	YARGAB_0.16_0		Yes	CO_2	Physisorption	Negligible	Potential
7_302	ZAFXAI		No	CO_2	Physisorption	Negligible	Yes
8_291	WANJIF		No	CO_2	Physisorption	Negligible	Yes
8_292	WANJIF		No	CO_2	Physisorption	Negligible	Yes
9_66	MALROJ_0.16_0		Yes	CO_2	Physisorption	Negligible	Yes
0_419	LAMGUB		No	H_2O	Physisorption	Negligible	No
0_426	OZAVES		No	H_2O	Physisorption	Negligible	No
0_441	FAHGAY		No	H_2O	Physisorption	Negligible	No
1_211	GUVZII_0.11_0		Yes	H_2O	Physisorption	Negligible	No
1_479	NARPAA		No	H_2O	Chemisorption	Negligible	No
2_418	XENMOU_SL		No	H_2O	Chemisorption	Negligible	No
2_432*	IRELAU		No	H_2O	Physisorption	Negligible	No
3_266	RIPKIM_0.08_1		Yes	H_2O	Physisorption	Negligible	No
3_387	MABJUV01		No	H_2O	Physisorption	Negligible	No

Continued on Next Page

Table S1 Continued from Previous Page

ID	MOF (CSD)	MOF (common name)	Defective	Adsorbate	Adsorption type	Deformation class	DAC favorability
4_145	ENISOK_0.12_0		Yes	H_2O	Physisorption	Negligible	No
4_148	KALFOU_0.16_0		Yes	H_2O	Physisorption	Negligible	Potential
4_167	KALFOU_0.16_0		Yes	H_2O	Physisorption	Negligible	Potential
4_494*	FARBIL	MOF-48	No	H_2O	Physisorption	Negligible	Potential
6_229	MIMSUY_0.08_1		Yes	H_2O	Physisorption	Negligible	Yes
6_430*	EKOPOK		No	H_2O	Physisorption	Negligible	Potential
8_203	CEGDUO_0.03_0		Yes	H_2O	Physisorption	Negligible	Potential
8_209	FAHGAY_0.12_0		Yes	H_2O	Physisorption	Negligible	Potential
8_226	QAVWAN_0.08_0		Yes	H_2O	Physisorption	Negligible	Potential
9_188	KEDJAG14_0.02_0		Yes	H_2O	Chemisorption	Negligible	Yes
9_444	VAGMAT	SNU-30	No	H_2O	Physisorption	Negligible	Potential
2_74	JOHSIJ_0.07_1		Yes	CO_2	Chemisorption	Significant	Yes
2_168	ECUDOX		No	CO_2	Chemisorption	Significant	Yes
3_78*,†	BETHIT_0.12_0		Yes	CO_2	Physisorption	Significant	No
6_70	KOFPEB04_0.08_0		Yes	CO_2	Chemisorption	Significant	Yes
6_73	KOFPEB04_0.08_0		Yes	CO_2	Physisorption	Significant	Yes
6_276	LUSHOX		No	CO_2	Chemisorption	Significant	Yes
7_94	MIMFOF_0.03_1	AIPO-BDA	Yes	CO_2	Chemisorption	Significant	Yes
9_289	RIPKIM		No	CO_2	Physisorption	Significant	Yes
0_217	WOBHEB_0.11_0		Yes	H_2O	Chemisorption	Significant	No
0_452*	SIVKAK		No	H_2O	Chemisorption	Significant	No
3_384	GOSDEZ		No	H_2O	Physisorption	Significant	No
3_402*	OVUCOY		No	H_2O	Chemisorption	Significant	No
4_469	LUKREO		No	H_2O	Chemisorption	Significant	No
5_85	OZAVES_0.12_0		Yes	H_2O	Chemisorption	Significant	No
5_361*	DEWRIH		No	H_2O	Chemisorption	Significant	No
6_210	EGEJIK_0.08_0		Yes	H_2O	Chemisorption	Significant	Potential
6_239	QUSSII_0.08_0		Yes	H_2O	Physisorption	Significant	Potential
7_245	ESEVIH_0.06_0		Yes	H_2O	Chemisorption	Significant	Potential

*MOF geometry initialized from ODAC23 re-relaxation initial positions.
†Omitted from all analyses due to pore collapse; see Figure S1.

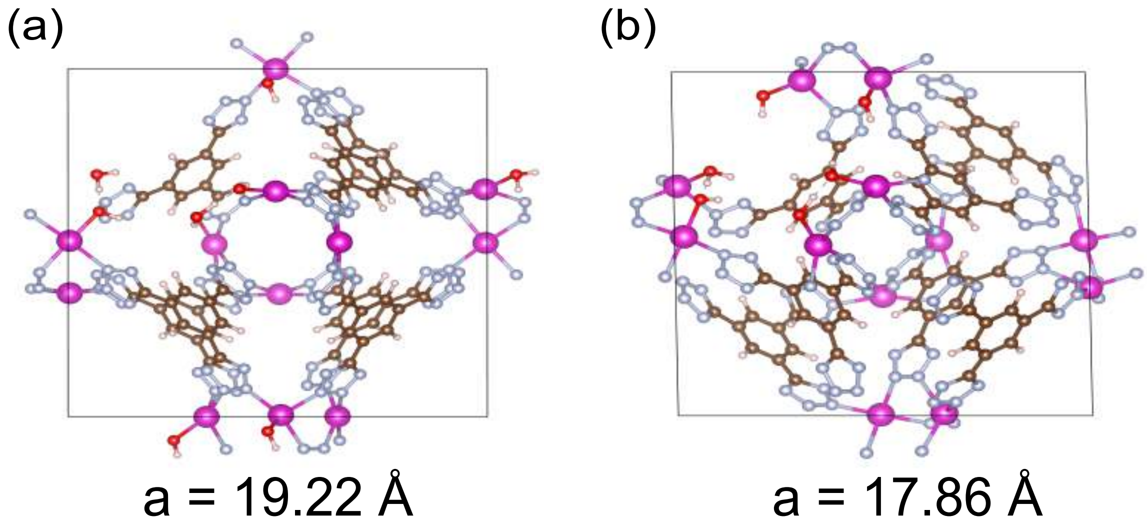

Figure S1: Relaxed empty MOF and corresponding lattice parameter, a, for system 3_78 (BETHIT_0.12_0) from (a) ODAC23 and (b) this work.

Table S2: Atomic charges for each atom in pristine DFT-relaxed ESURER03 (system 2_173); the other CoRE MOFs used in this study flagged as "charged" by Jin et al.[86] are QOPCEE, FAHGAY, JOHSIJ, ECUDOX, GOSDEZ, OVUCOY, PIWSEV, DEWRIH, MIMFOF, YARGAB, ZAFXAI, and KEDJAG14

Atom no.	Element	EQeq charge [e]	DDEC charge [e]	Atom no.	Element	EQeq charge [e]	DDEC charge [e]
1	Co	1.141	0.731	75	C	–0.096	–0.158
2	Co	1.164	0.732	76	C	0.018	–0.097
3	Co	1.073	0.731	77	C	–0.065	-0.042
4	Co	1.047	0.732	78	C	–0.036	-0.153
5	Co	1.139	0.732	79	C	0.340	0.582
6	Co	1.118	0.732	80	C	0.073	0.134
7	Co	1.048	0.732	81	C	0.058	0.130
8	Co	1.079	0.732	82	C	–0.088	–0.157
9	Co	1.202	0.817	83	C	0.021	–0.097
10	Co	1.144	0.817	84	C	–0.054	-0.042
11	Co	1.206	0.816	85	C	–0.033	-0.153
12	Co	1.153	0.817	86	C	0.340	0.582
13	H	0.042	0.119	87	C	0.083	0.134
14	H	0.025	0.106	88	C	–0.001	0.130
15	H	–0.018	0.129	89	C	0.194	–0.158
16	H	0.186	0.386	90	C	–0.070	–0.097
17	H	0.037	0.119	91	C	–0.030	-0.042
18	H	0.013	0.107	92	C	–0.068	-0.153
19	H	–0.024	0.129	93	C	0.341	0.582
20	H	0.178	0.386	94	C	0.076	0.134
21	H	–0.026	0.119	95	C	0.002	0.130
22	H	–0.004	0.106	96	C	0.191	–0.157
23	H	0.045	0.129	97	C	–0.073	–0.097
24	H	0.155	0.386	98	C	–0.038	–0.042
25	H	–0.026	0.119	99	C	–0.071	–0.153
26	H	0.007	0.106	100	C	0.338	0.582
27	H	0.049	0.129	101	N	–0.234	–0.251
28	H	0.163	0.386	102	N	–0.176	0.017
29	H	0.033	0.119	103	N	–0.221	–0.230
30	H	0.027	0.107	104	N	–0.243	–0.251
31	H	0.112	0.129	105	N	–0.210	0.018
32	H	0.181	0.386	106	N	–0.234	–0.230
33	H	0.039	0.119	107	N	–0.235	–0.251
34	H	0.039	0.107	108	N	–0.224	0.017
35	H	0.117	0.129	109	N	–0.257	–0.230
36	H	0.188	0.386	110	N	–0.230	–0.251
37	H	–0.143	0.119	111	N	–0.211	0.018
38	H	0.102	0.107	112	N	–0.251	–0.230
39	H	0.052	0.129	113	N	–0.190	–0.251
40	H	0.214	0.386	114	N	–0.218	0.017
41	H	–0.146	0.119	115	N	–0.239	–0.230
42	H	0.092	0.107	116	N	–0.181	–0.250
43	H	0.048	0.129	117	N	–0.198	0.017
44	H	0.206	0.386	118	N	–0.227	–0.230
45	C	0.014	0.134	119	N	–0.223	–0.250
46	C	0.069	0.130	120	N	–0.171	0.017
47	C	–0.110	0.–0.157	121	N	–0.159	–0.230
48	C	0.032	–0.097	122	N	–0.229	–0.250
49	C	–0.113	–0.043	123	N	–0.198	0.018
50	C	–0.034	–0.153	124	N	–0.166	–0.230
51	C	0.362	0.582	125	O	–0.718	–0.782
52	C	0.004	0.134	126	O	–0.502	–0.536
53	C	0.065	0.130	127	O	–0.448	–0.496
54	C	–0.118	–0.158	128	O	–0.781	–0.782
55	C	0.028	–0.097	129	O	–0.525	–0.536
56	C	–0.124	–0.041	130	O	–0.478	–0.497
57	C	–0.038	–0.153	131	O	–0.749	–0.782
58	C	0.358	0.582	132	O	–0.443	–0.537
59	C	0.030	0.134	133	O	–0.448	–0.496
60	C	0.033	0.130	134	O	–0.678	–0.782
61	C	–0.124	–0.158	135	O	–0.420	–0.536
62	C	0.009	–0.097	136	O	–0.401	–0.497
63	C	–0.095	–0.042	137	O	–0.719	–0.782
64	C	–0.047	–0.153	138	O	–0.354	–0.537

Continued on Next Page

Table S2 Continued from Previous Page

Atom no.	Element	EQeq charge [e]	DDEC charge [e]	Atom no.	Element	EQeq charge [e]	DDEC charge [e]
65	C	0.350	0.582	139	O	–0.430	–0.497
66	C	0.036	0.133	140	O	–0.638	–0.782
67	C	0.033	0.130	141	O	–0.331	–0.536
68	C	–0.118	–0.157	142	O	–0.381	–0.497
69	C	0.010	–0.097	143	O	–0.634	–0.782
70	C	–0.085	–0.042	144	O	–0.442	–0.536
71	C	–0.045	–0.154	145	O	–0.424	–0.497
72	C	0.348	0.582	146	O	–0.690	–0.782
73	C	0.064	0.134	147	O	–0.462	–0.536
74	C	0.054	0.130	148	O	–0.452	–0.497

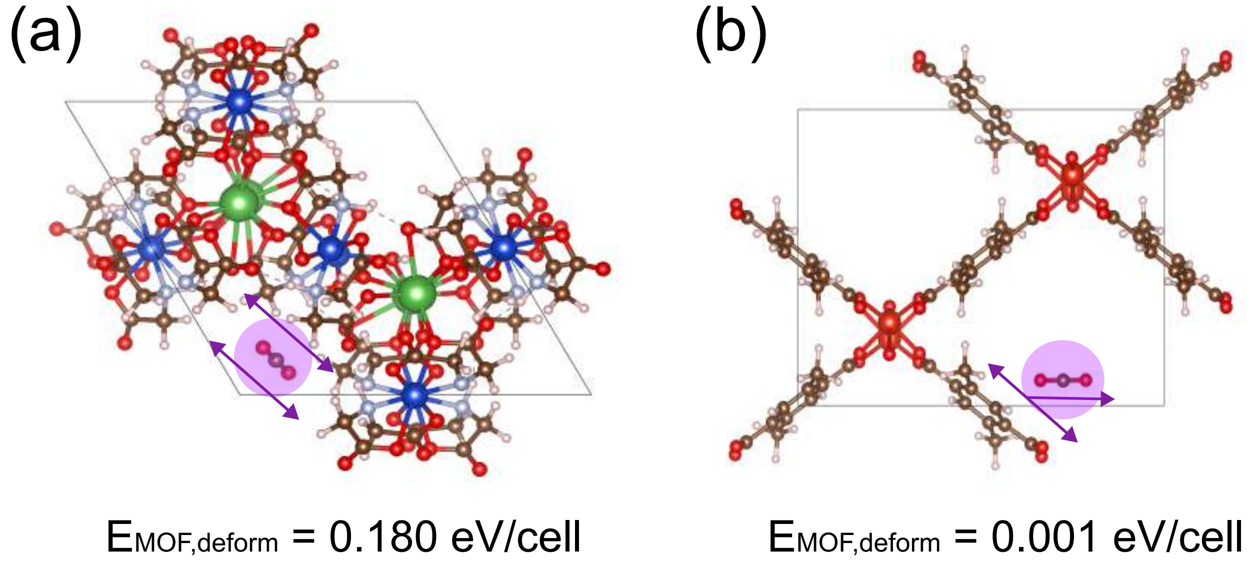

Figure S2: DFT–relaxed MOF+adsorbate configuration for (a) system 6_73 (CO_2 adsorption in KOFPEB04_0.08_0) and (b) system 4_194 (CO_2 adsorption in FARBIL) with the corresponding MOF deformation energies. The CO_2 molecules are shaded in purple, and the purple lines indicate whether the CO_2 aligns parallel to or semi-orthogonal to the pore surface.

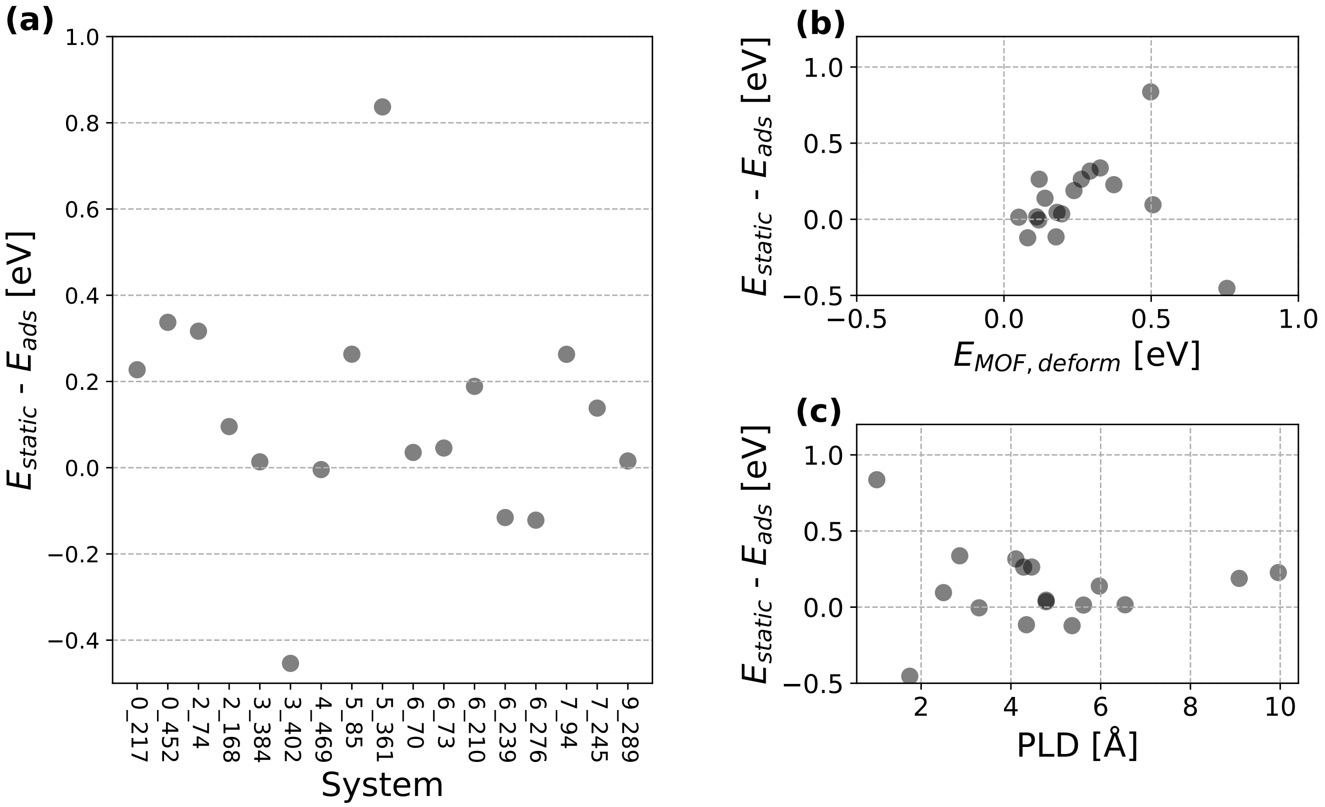

Figure S3: (a) Errors between E_{static} and E_{ads} using DFT for 17 systems which undergo significant MOF deformation. (b) Comparison of errors to DFT MOF deformation energies. (c) Comparison of errors to pore limiting diameter (PLD). In all subplots, the y-axis is limited to [-0.5, 1.0] eV for viewing, and points outside this range are omitted.

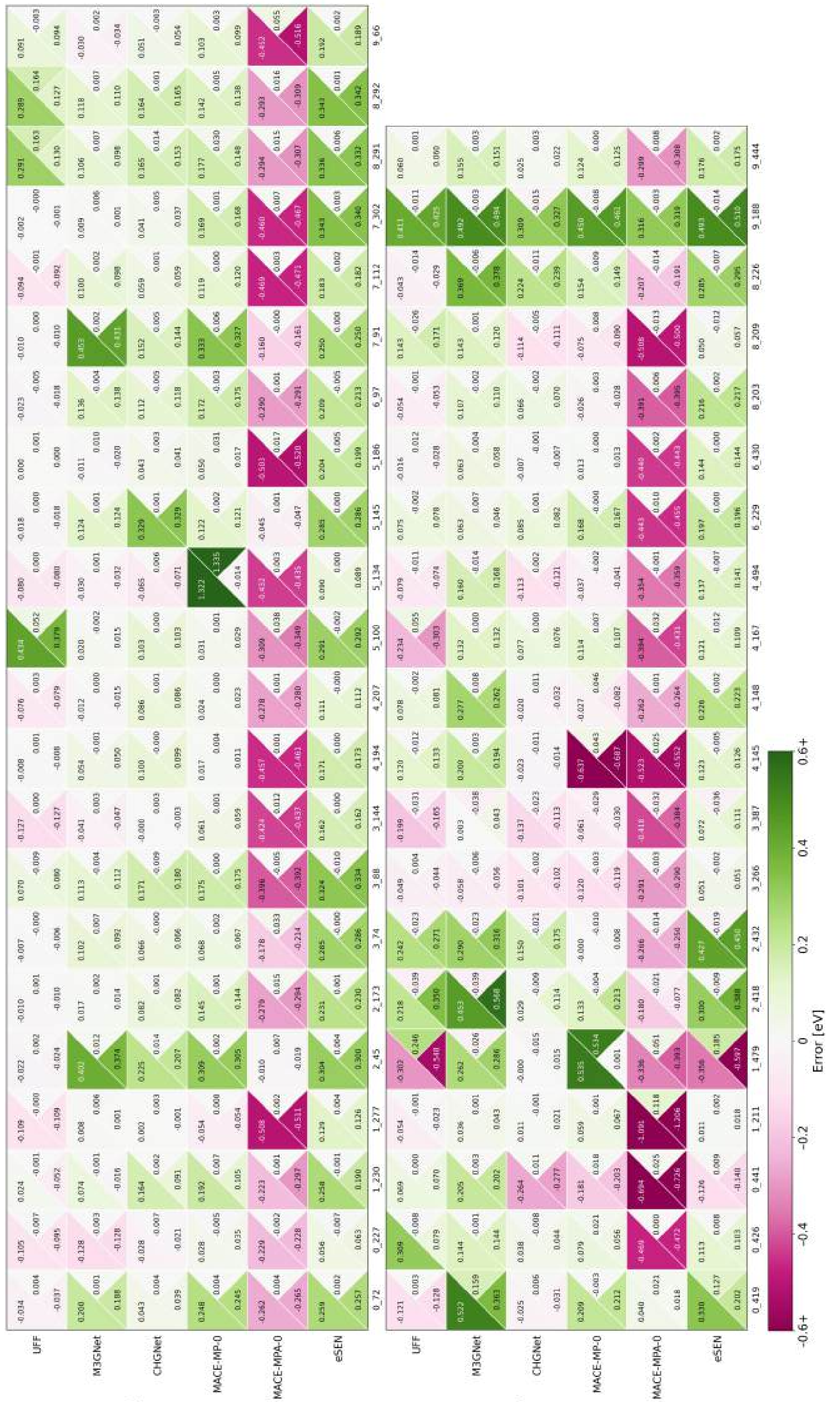

Figure S4: Adsorption energy errors (upper left triangle), interaction energy errors (bottom triangle), and MOF deformation errors (right triangle) from FF relaxations for systems that undergo negligible deformation separated by adsorbate. All energies are in eV using the color bar shown in the figure.

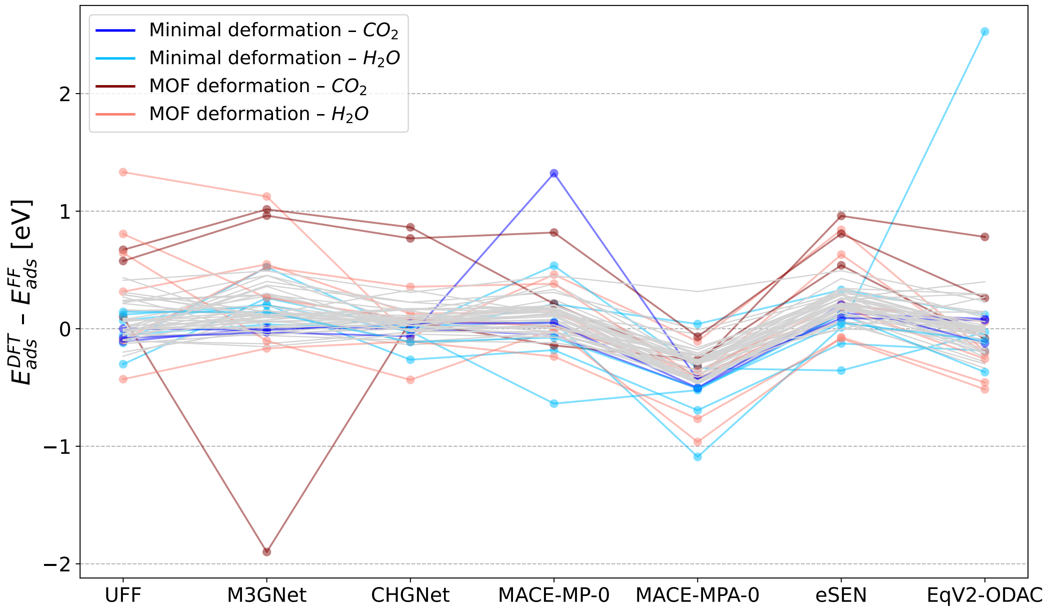

Figure S5: Adsorption energy errors where errors for one MOF+adsorbate system are connected by lines to aid visualization. Systems with errors less than 0.2 eV for all seven FFs are excluded for simplicity. Plots are bound on the y-axis from [-2,2.5] eV for scaling purposes.

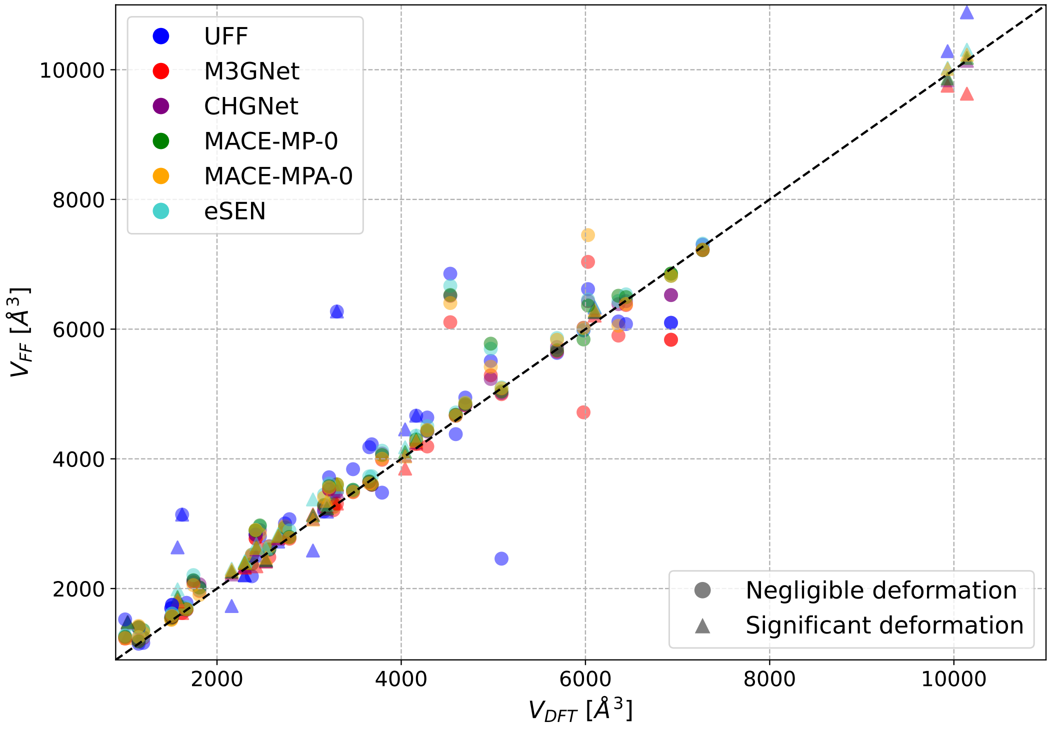

Figure S6: Parity plot of volumes per unit cell determined by DFT and FF relaxation. Colors denote the FF used for the y-axis, and shapes denote whether the system undergoes significant deformation according to DFT.

Table S3: Computational cost of performing all relaxations outlined in this work for each model in CPU hours. Calculations were performed using Intel Xeon Gold 6226 processors.

Model	Cost (CPU hours)
DFT	44,444.4
UFF	68.1
M3GNet	13.7
CHGNet	9.4
MACE-MP-0	94.7
MACE-MPA-0	120.0
eSEN	202.7
EqV2-ODAC	80.5

Table S4: R^2 and Pearson correlation coefficients (r) for FF E_{ads} predictions relative to DFT. The scikit-learn implementation for R^2 was used as defined by Eqn. 8. R^2 is negative when the FF predictions are worse than predicting the mean of the DFT adsorption energies

FF	R^2	r
UFF	–0.648	0.540
M3GNet	–2.062	0.152
CHGNet	0.248	0.648
MACE-MP-0	–0.507	0.549
MACE-MPA-0	–2.044	0.631
eSEN	–0.786	0.477
EqV2-ODAC	–1.838	0.540

Methods for gas-phase adsorbate energy corrections

To validate that gas-phase errors did not significantly affect conclusions presented in the main text, we used a regression approach to determine gas-phase corrections as shown in Table S6. For each FF in Table S6, linear regression was used to compute a correction to the gas-phase CO_2 and H_2O molecular energies such that the errors between DFT E_{ads} and FF E_{ads} were minimized. The regression was done using the scikit-learn Python package.[91] The regression problem is formally defined as

$$d = x + \mathbf{M}b \tag{9}$$

where d is a 59×1 vector containing the DFT-calculated E_{ads} for each system, x is a 59×1 vector of FF-calculated E_{ads}, $\mathbf{M}$ is a 59×2 matrix encoding whether a system contains CO_2 or H_2O, and b is a 2×1 vector of corrections to be computed by the regression. The feature matrix $\mathbf{M}$ contains only 0 and 1, with a 1 in the first column indicating a CO_2 system and a 1 in the second column indicating a H_2O system. The other column is always 0. This in effect creates two distinct linear regression problems, resulting in one correction for CO_2 that minimizes the error between DFT and FF for the CO_2 systems, and likewise for H_2O. A separate regression problem was solved for each FF, so each pair of corrections is different as shown in Table S6.

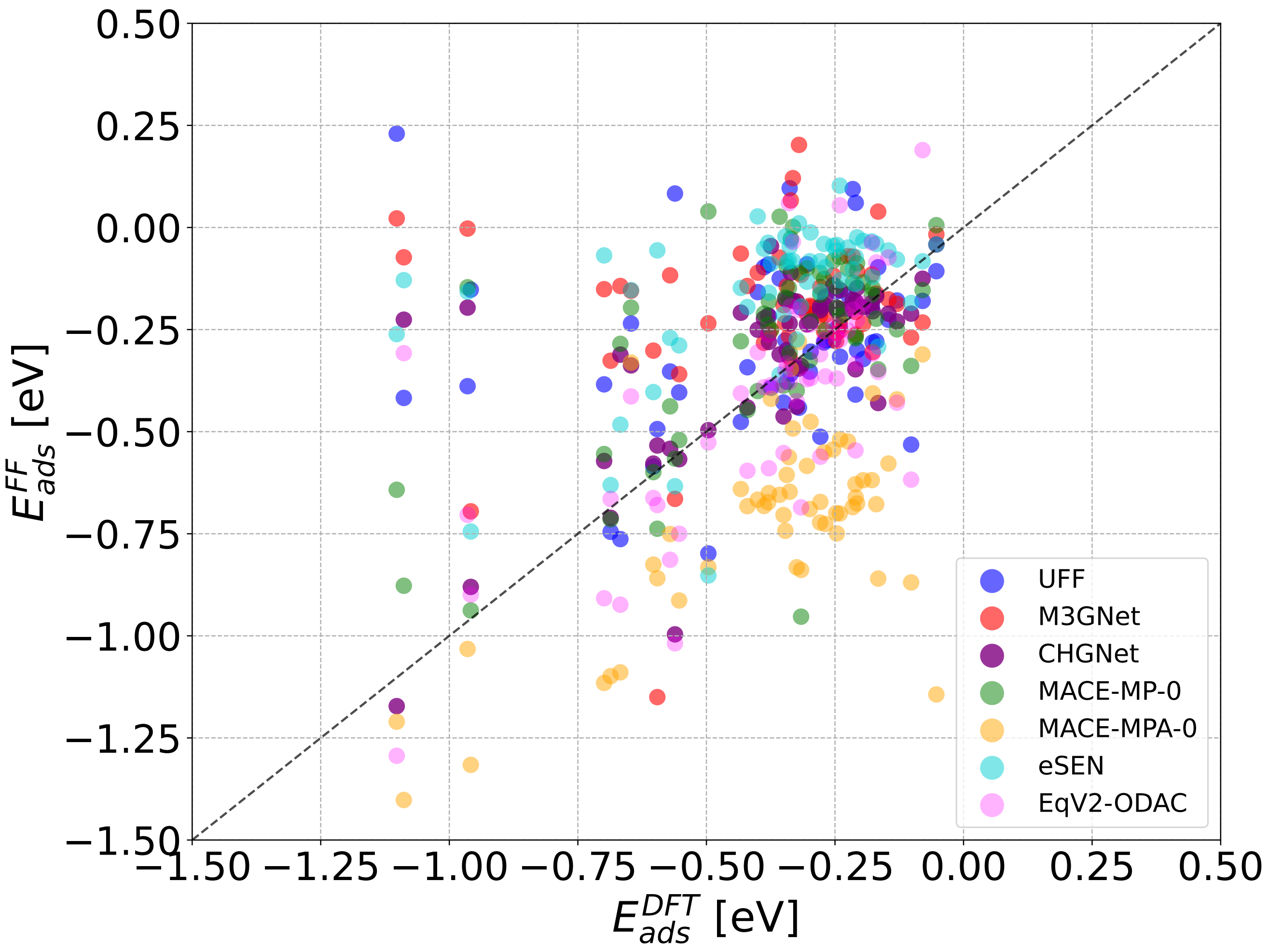

Figure S7: Comparison of DFT E_{ads} with FF E_{ads} predictions for all models tested. Points falling outside the [-1.5, 0.5] eV range are excluded.

Table S5: FF errors for select systems with short distances between MOF and adsorbate in the DFT-relaxed geometry

System	Shortest distance between MOF and adsorbate [Å]	UFF E_{ads} error [eV]	CHGNet E_{ads} error [eV]	MACE-MP-0 E_{ads} error [eV]
2_74	1.926	0.671	0.863	0.211
4_469	1.608	–0.096	0.356	0.382
7_94	1.970	0.576	0.768	0.818

Table S6: E_{ads} Error statistics for MLFFs using corrected gas-phase adsorbate energies ($E_{adsorbate}$)

FF	CO_2 $E_{adsorbate}$ correction [eV]	H_2O $E_{adsorbate}$ correction [eV]	MAE with direct $E_{adsorbate}$ [eV]	MAE with corrected $E_{adsorbate}$ [eV]	R^2 with corrected $E_{adsorbate}$
UFF	–0.082	–0.116	0.186	0.197	–0.460
M3GNet	–0.064	–0.245	0.247	0.196	–1.458
CHGNet	–0.135	–0.006	0.124	0.113	0.414
MACE-MP-0	–0.176	–0.068	0.181	0.160	–0.180
MACE-MPA-0	0.305	0.385	0.357	0.139	0.200
eSEN	-0.271	-0.174	0.243	0.143	0.169
